# Comparative analysis of the chloroplast and mitochondrial genomes of *Saposhnikovia divaricata* revealed the possible transfer of plastome repeat regions into the mitogenome

**DOI:** 10.1186/s12864-022-08821-0

**Published:** 2022-08-10

**Authors:** Yang Ni, Jingling Li, Haimei Chen, Jingwen Yue, Pinghua Chen, Chang Liu

**Affiliations:** 1 Key Laboratory of Bioactive Substances and Resource Utilization of Chinese Herbal Medicine from Ministry of Education, Engineering Research Center of Chinese Medicine Resources from Ministry of Education, Center for Bioinformatics, Institute of Medicinal Plant Development, Chinese Academy of Medical Sciences, Peking Union Medical College, No. 151, Malianwa North Road, Haidian District, 100193 Beijing, P. R. China; 2 College of Agriculture, Fujian Agriculture and Forestry University, No.15, Shang Xiadian Road, Fuzhou, Fujian Province 350002 P. R. China

**Keywords:** De novo assembly, Organelle genomes, Phylogenetic analysis, DNA transfer, Selective pressure analysis

## Abstract

**Background:**

*Saposhnikovia divaricata* (Turcz.) Schischk. is a perennial herb whose dried roots are commonly used as a source of traditional medicines. To elucidate the organelle-genome-based phylogeny of *Saposhnikovia* species and the transfer of DNA between organelle genomes, we sequenced and characterised the mitochondrial genome (mitogenome) of *S. divaricata*.

**Results:**

The mitogenome of *S. divaricata* is a circular molecule of 293,897 bp. The nucleotide composition of the mitogenome is as follows: A, 27.73%; T, 27.03%; C, 22.39%; and G, 22.85. The entire gene content is 45.24%. A total of 31 protein-coding genes, 20 tRNAs and 4 rRNAs, including one pseudogene (*rpl*16), were annotated in the mitogenome. Phylogenetic analysis of the organelle genomes from *S. divaricata* and 10 related species produced congruent phylogenetic trees. Selection pressure analysis revealed that most of the mitochondrial genes of related species are highly conserved. Moreover, 2 and 46 RNA-editing sites were found in the chloroplast genome (cpgenome) and mitogenome protein-coding regions, respectively. Finally, a comparison of the cpgenome and the mitogenome assembled from the same dataset revealed 10 mitochondrial DNA fragments with sequences similar to those in the repeat regions of the cpgenome, suggesting that the repeat regions might be transferred into the mitogenome.

**Conclusions:**

In this study, we assembled and annotated the mitogenome of *S. divaricata*. This study provides valuable information on the taxonomic classification and molecular evolution of members of the family Apiaceae.

**Supplementary Information:**

The online version contains supplementary material available at 10.1186/s12864-022-08821-0.

## Background

*Saposhnikovia divaricata* (Turcz.) Schischk. (http://www.theplantlist.org/tpl1.1/record/kew-2480406; last accessed on April 7, 2022) is a perennial herb whose dried roots have been commonly used as traditional medicines over the past 2000 years [1]. *S. divaricata* belongs to the family Apiaceae [2]*.* It is mainly found in China, Japan, Korea and other Asian countries [1]. In China, *S. divaricata* is widely cultivated in Henan, Jiangsu, Shaanxi, Hebei and Shandong provinces [3]. *S. divaricata* is resistant to salinity, cold and drought, and it is often grown as a sand-fixing plant in the dry areas of northwest China [4]. Moreover, *S. divaricata* is usually used as a medicine to treat colds, arthralgia, headaches and other diseases. Thus far, over 100 compounds have been isolated from *S. divaricata,* including abundant chromones, coumarins, acid esters and polyacetylenes, which are potential active components for the treatment of diseases of the immune, nervous and respiratory systems [5]. Exploring the organelle genomes of *S. divaricata* will help us classify *Saposhnikovia* species and provide a genetic resource for further study.

Organelle genomes are critical in sustaining an organism’s growth and development. Like the nuclear genome, the plant organelle genome has various strategies to repair DNA damage and maintain the integrity of the genetic material to withstand the damage caused by genotoxic stresses [6]. Organelle genomes have been extensively analysed to understand a taxon’s classification and evolution. To date, 6804 complete chloroplast genomes (cpgenomes) and 433 plant mitochondrial genomes (mitogenomes) have been released in the GenBank Organelle Genome database (https://www.ncbi.nlm.nih.gov/genome/browse/; last accessed on February 25, 2022) [7]. The number of sequenced mitogenomes is fewer than that of the cpgenomes probably because of the complex structures of the former, which resulted from the violent redox reactions that accompanied the rearrangement of some DNA fragments [8, 9]. Previous studies have found that the evolution of the mitogenome affects cytoplasmic male sterility (CMS), a phenomenon with important implications to plant breeding genetics [10].

The cpgenome of *S. divaricata* has been reported in a previous study [11]. However, no study has described its mitogenome and the exchange of DNA between the cpgenome and the mitogenome. In this study, we de novo assembled the cpgenome and the mitogenome of *S. divaricata*. We report the mitogenome of this species for the first time and compared the differences between the cpgenome assembled herein and the one published in a previous study. Moreover, we systematically analysed the gene content, repeat sequences, selective pressure and RNA-editing sites. Finally, we explored the phylogenetic relationships among *S. divaricata* and 10 related species. This study provides valuable information on the taxonomic classification, molecular evolution and breeding of *Saposhnikovia* species.

## Results

### General features of the organelle genomes of *S. divaricata*

**Fig. 1:**
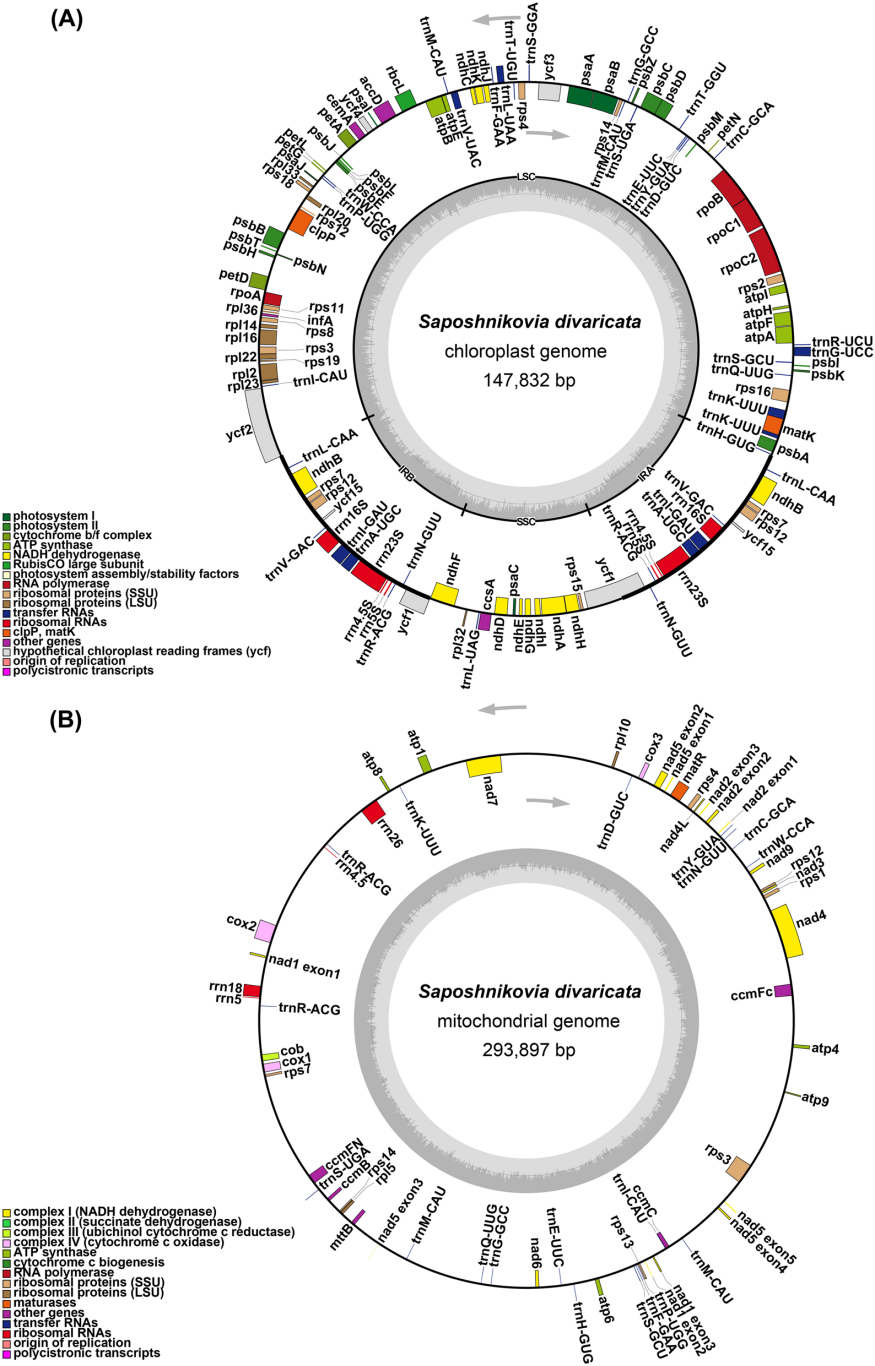
The circular maps of the organelle genomes of *S. divaricata*. **A** The circular map of the cpgenome. **B** The circular map of the mitogenome. Genomic features transcribed clockwise and counter-clockwise are drawn on the inside and outside of the circle, respectively. Genes are color-coded based on their functional groups. GC content is represented on the inner circle by the dark gray plot

The cpgenome (MZ089852) is 147,832 bp and has a typical quadripartite structure consisting of a pair of inverted repeats (IR) regions of 18,653 bp, a large single-copy (LSC) region of 93,202 bp and a small single-copy (SSC) region of 17,324 bp (Fig. 1A). The gene contents (GC) of the IR, LSC and SSC regions are 44.58, 35.94 and 30.85%, respectively. The cpgenome encodes 114 unigenes, including 80 protein-coding genes, 30 tRNAs and 8 rRNA genes (Table S1). Eighteen genes have one intron (*trn*K-UUU, *rps*16, *trn*G-UCC, *atp*F, *rpo*C1, *trn*L-UAA, *trn*V-UAC, *pet*B, *pet*D, *rpl*16, *rpl*2, *ndh*B, *trn*I-GAU, *trn*A-UGC, *ndh*A, *trn*A-UGC, *trn*I-GAU and *ndh*B), and two genes (*ycf*3 and *clp*P) contain two introns (Table S2, Figures S1 and S2). The GCs of the coding sequences, tRNAs and rRNA sequences are 37.95, 53.55 and 55.22%, respectively. We then compared the cpgenome and the one published before using dotplot. The results showed that the two cpgenomes were highly collinear (Figure S3), and differed in two one-base indels (Figure S4).

Several mitogenomes of Apiaceae have been previously reported, which are from *Daucus carota* subsp*. sativus* (281,132 bp) and *Bupleurum falcatum* (463,792 bp) [12, 13]. The *S. divaricata* mitogenome (MZ128146) is a circular molecule of 293,897 bp (Fig. 1B). The nucleotide composition of the whole mitogenome is as follows: A, 27.73%; T, 27.03%; C, 22.39%; and G, 22.85. The entire GC is 45.24%, similar to that of *D. carota* subsp*. sativus* (45.41%). A total of 31 protein-coding genes, 20 tRNAs and 4 rRNA genes, including one pseudogene (*rpl*16), were annotated in the mitogenome (Table 1). There were eight genes contain introns and the composition of introns were shown in the Table S3.

**Table 1:** Gene composition in the mitogenome of *S. divaricata*

Group of genes	Name of genes
ATP synthase	*atp*1, *atp*4, *atp*6, *atp*8, *atp*9
Cytochrome c biogenesis	*ccm*B, *ccm*C, *ccm*Fc^b^, *ccm*Fn
Ubichinol cytochrome c reductase	*Cob*
Cytochrome c oxidase	*cox*1^b^, *cox*2^b^, *cox*3
Maturases	*mat*R
Transport membrane protein	*mtt*B
NADH dehydrogenase	*nad*1^b^, *nad*2^b^, *nad*3, *nad*4^b^, *nad*4L, *nad*5^b^, *nad*6, *nad*7^b^, *nad*9
Large subunit of ribosomal proteins	*rpl*5, *rpl*10, *rpl*16^a^
Small subunit of ribosomal proteins	*rps*3, *rps*4, *rps*12, *rps*13, *rps*14
Ribosomal RNAs	*rrn*4.5,*rrn*5, *rrn*18, *rrn*26
Transfer RNAs	*trn*Y-GUA, *trn*W-CCA, *trn*S-UGA, *trn*S-GCU, *trn*Q-UUG, *trn*P-UGG, *trn*N-GUU, *trn*M-CAU, *trn*K-UUU, *trn*I-CAU, *trn*H-GUG, *trn*G-GCC, *trn*F-GAA, *trn*E-UUC, *trn*D-GUC, *trn*C-GCA, *trn*M-CAU, *trn*S-UGA, *trn*R-ACG(×2)

‘^a^’ Labeled the pseudogenes, ‘^b^’ Labeled the genes that contain introns

### Repeat analysis

**Fig. 2:**
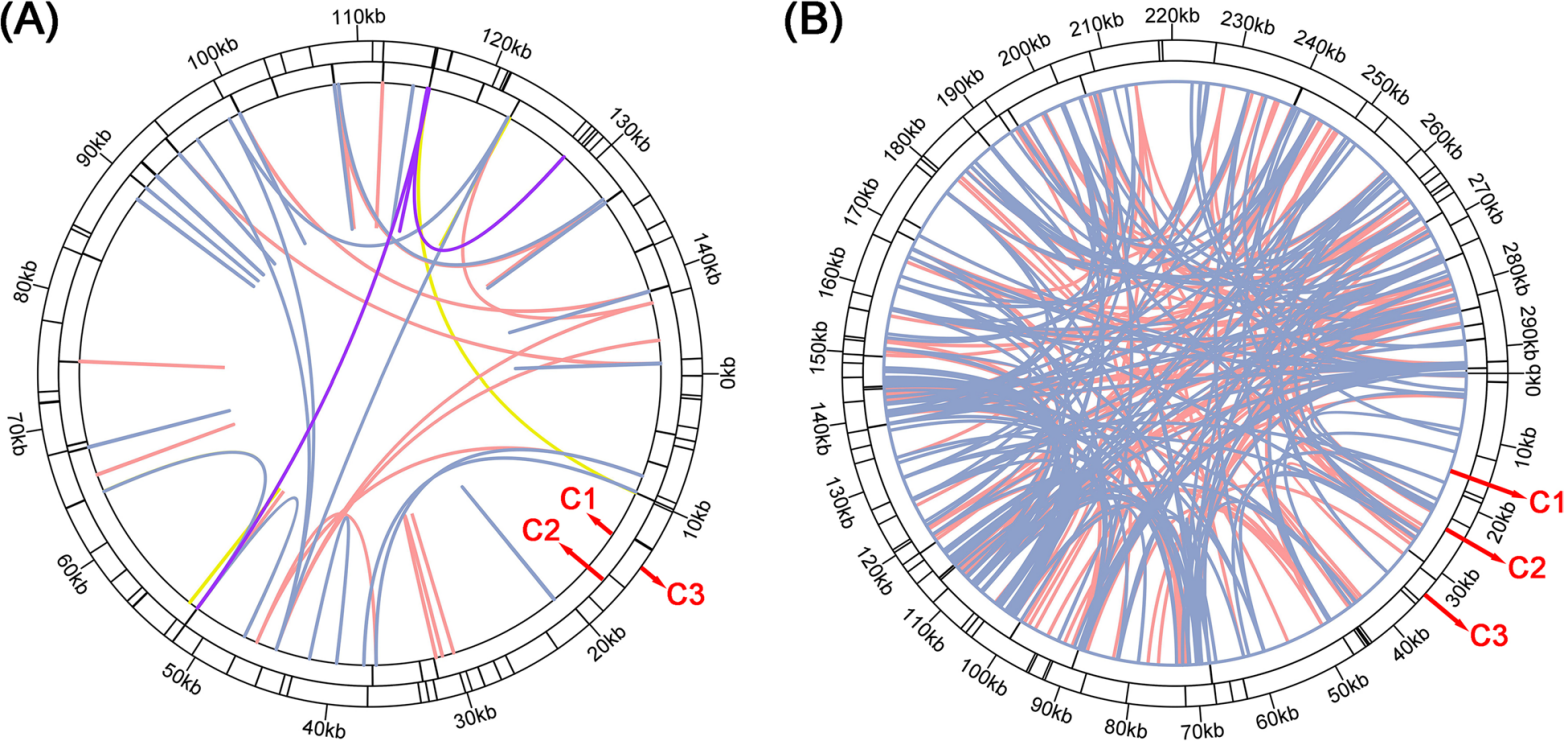
The Repeat analysis of the *S. divaricata* organelle genomes. **A** The repeat sequences identified in the cpgenome. **B** The repeat sequences identified in the mitogenome. The C1 circle shows the dispersed repeats connected with yellow, blue, purple, and pink arcs from the center going outward. The C2 circle shows the tandem repeats as short bars. The C3 circle shows the microsatellite sequences identified using MISA. The scale is shown on the C3 circle, with intervals of 10 kb

Microsatellites are also known as simple sequence repeats (SSRs). They are mono-, di-, tri-, tetra- or pentanucleotide DNA units and mostly appear in eukaryotes [14]. A total of 76 and 41 SSRs were detected in the cpgenome and the mitogenome, respectively (Fig. 2A, Tables S4 and S5). In the cpgenome, the most abundant SSRs have a single-nucleotide repeat unit, particularly A/T. The number of A/T repeat units accounts for 88.4% of all identified SSR repeats. However, the SSRs are evenly distributed among the various types in the mitogenome. A total of 12, 4, 6, 16 and 3 SSRs have mono-, di-, tri-, tetra- and pentanucleotide repeat units, respectively. The most abundant SSRs in the mitogenome have a tetranucleotide repeat unit, representing 39.0% of all the repeat number. These SSRs could be potential identification markers for *S. divaricata*.

Tandemly repeated DNA sequences, which have a unit length longer than 6 bp, are highly dynamic components of genomes [15]. Most repeats are found in intergenic regions, but some are in coding sequences or pseudogenes [16] (Fig. 2B, Tables S6 and S7). A total of 25 and 26 tandem repeats were identified in the cpgenome and the mitogenome, respectively, and these repeats were evaluated further for their potential application in DNA fingerprinting.

Dispersed repeats are essential in generating genetic diversity, and they make valuable contributions to the evolution of plant genomes [17]. There are four kinds of dispersed repeats, namely, forward repeats, reverse repeats, complement repeats and palindromic repeats. In the cpgenome, all four types of dispersed repeats were found. In both genomes, the most abundant and the longest repeats are forward repeats, with the longest fragment being 22,397 bp in the mitogenome. Its number accounts for 34.7% of the total repeats in the mitogenome (Fig. 2, Tables S8 and S9). By contrast, only 33 forward repeats and 24 palindromic repeats were found in the cpgenomes, and most of them are 30–50 bp long.

### Sequence similarity between the mitogenome and the cpgenome

**Fig. 3:**
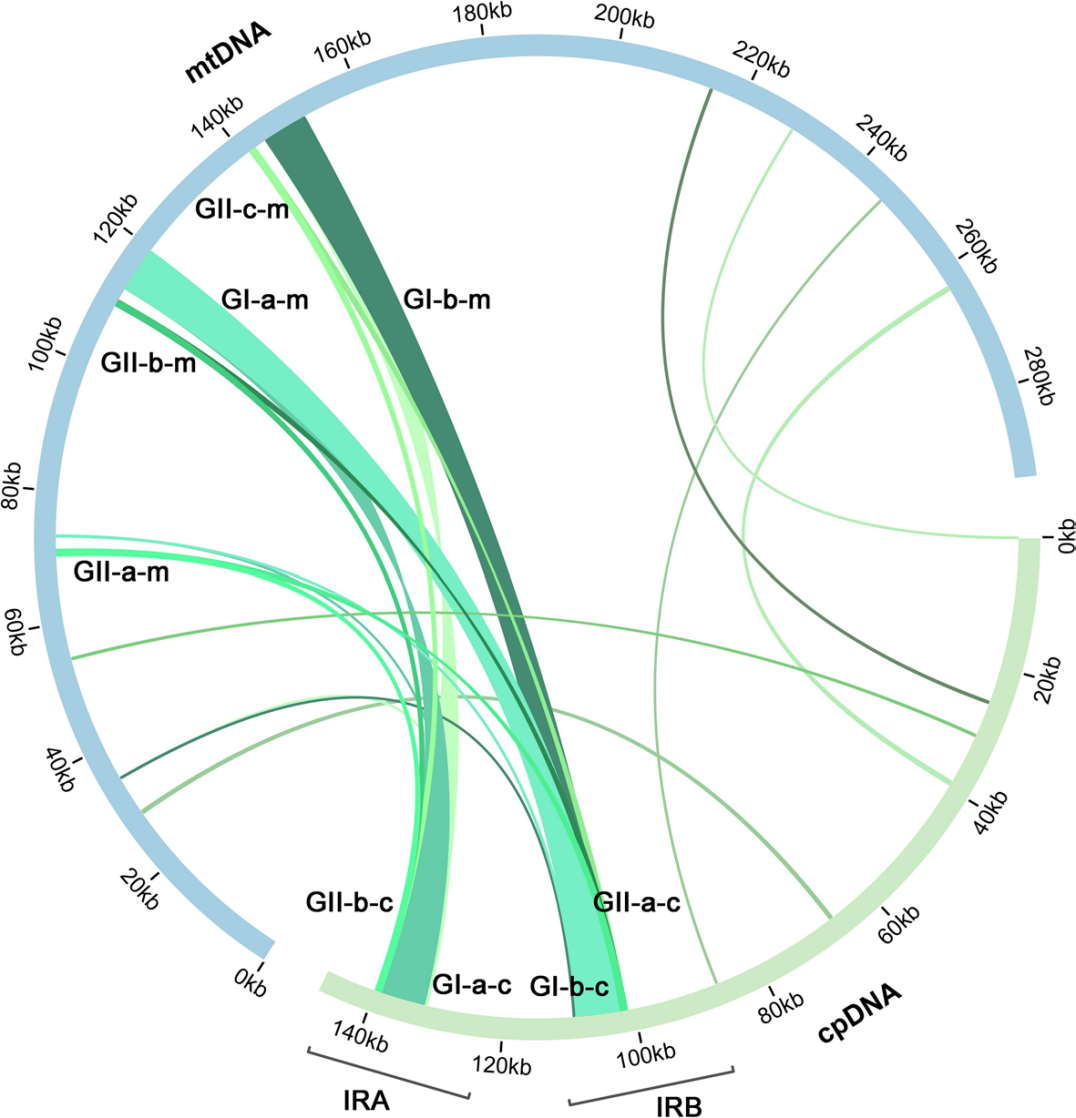
Comparison of the cpgenome and mitogenome of *S. divaricata*. The blue and green outer arcs represent the mitogenome (mtDNA) and cpgenome (cpDNA), respectively, and the inner green arcs show the homologous DNA fragments. The scale is shown on the outer arcs, with intervals of 20 kb. The repeat sequences in groups I and II are shown. The sequence name ‘GI-a-m’ indicates that this sequence belongs to Group I, repeat sequence ‘a’ in the mitogenome (‘m’). GI represent the group I. The letter in the middle represent the sequence identifier in the same group. The letter at the end indicates whether the sequence is a segment of the cpgenome or the mitogenome

A total of 10 groups of mitogenome fragments were identified to likely be derived from the cpgenome according to sequence similarity (Fig. 3, Table S10). These fragments add up to 17,921 bp in length and occupy 6.1% of the mitogenome. We numbered the group from ‘I’ to ‘X’. Group I contain two repetitive sequences of 6813 bp long (GI-a-m: 119569–112,758; Gl-b-m: 150885–144,074). Their sequences are similar to those in the IR regions of the cpgenome (Gl-a-c: 101995–108,807; Gl-b-c: 139040–132,228). Group II contains three repeat sequences in the mitogenome (GII-a-m: 71079–70,221; GII-b-m: 110682–109,824; GII-c-m: 141998–141,140). Their sequences are also similar to those in the IR regions of the cpgenome. Groups III and IV contain unique sequences of 104 and 82 bp respectively (GIII-m: 73257–73,155; GIV-m: 34749–34,668), similar to the sequences in the IR regions of the cpgenome (Table S10). The six other groups contain only single-copy sequences in the mitogenome and the cpgenome, and they represent 8.063% of the entire homologous DNA sequences between the two genomes. For the repeat direction, if the repeat sequences were in the protein-coding region, we used the sequences in the sense strand. However, if the repeat sequences were in the noncoding regions, we did not specify the direction (Table S10). These similar sequences might have resulted from the transfer of plastome sequences into the mitogenome during evolution.

### Phylogenetic analysis

**Fig. 4:**
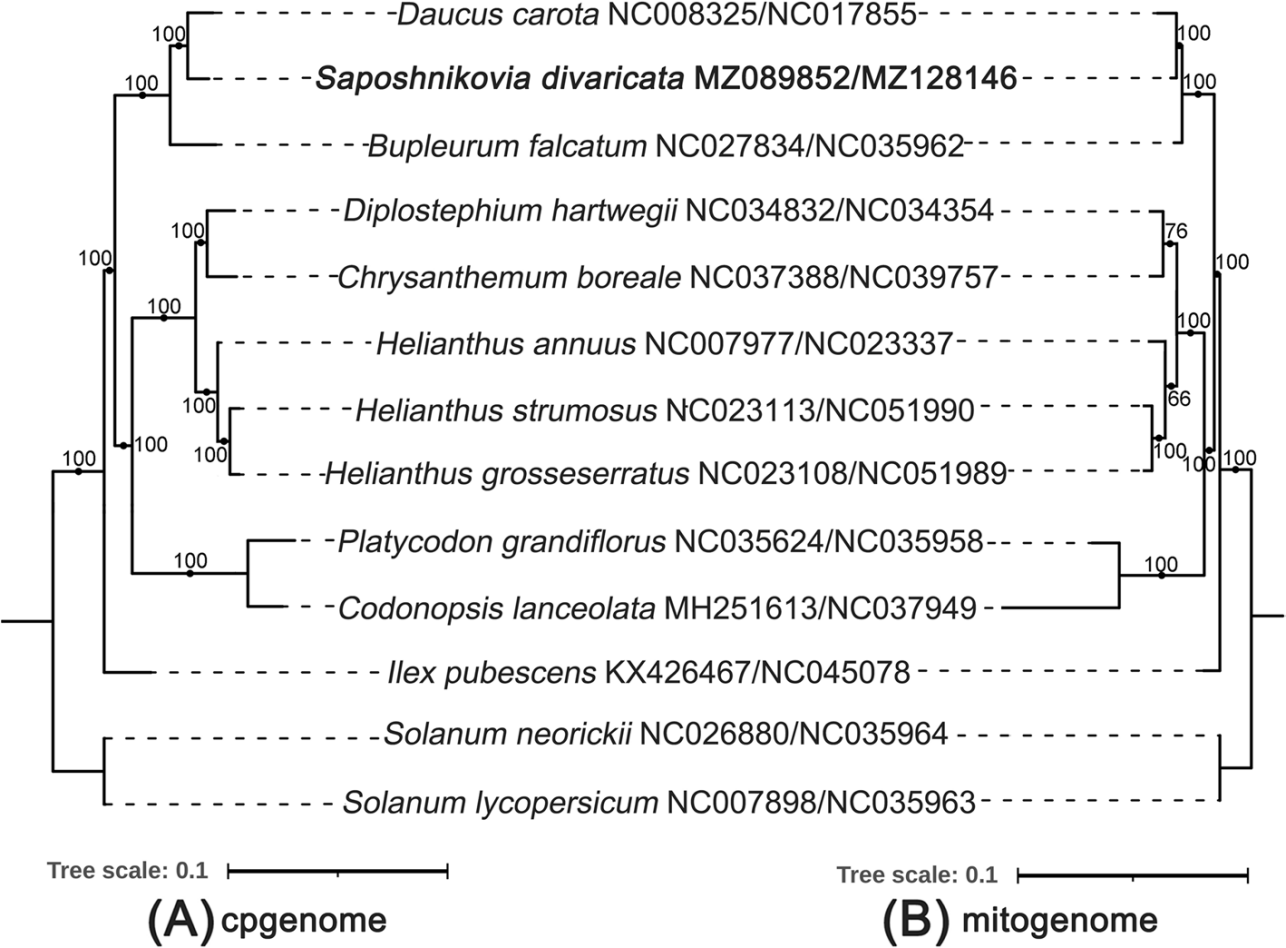
The phylogenetic relationships between *S. divaricata* and other 10 related plants. **a** phylogenetic analysis of cpgenomes based on the nucleotide sequences of 71 protein-coding genes from the cpgenome (*atp*A, *atp*B, *atp*E, *atp*F, *atp*H, *ccs*A, *cem*A, *mat*K, *ndh*A, *ndh*B, *ndh*C, *ndh*D, *ndh*E, *ndh*F, *ndh*G, *ndh*H, *ndh*I, *ndh*J, *ndh*K, *pet*A, *pet*D, *pet*G, *pet*L, *pet*N, *psa*A, *psa*B, *psa*C, *psa*I, *psa*J, *psb*A, *psb*B, *psb*C, *psb*D, *psb*E, *psb*F, *psb*H, *psb*I, *psb*J, *psb*K, *psb*L, *psb*M, *psb*N, *psb*T, *rbc*L, *rpl*14, *rpl*16, *rpl*20, *rpl*22, *rpl*2, *rpl*32, *rpl*33, *rpl*36, *rpo*A, *rpo*B, *rpo*C1, *rpo*C2, *rps*11, *rps*12, *rps*14, *rps*15, *rps*16, *rps*18, *rps*19, *rps*2, *rps*3, *rps*4, *rps*7, *rps*8, *ycf*2, *ycf*3, *ycf*4). **b** phylogenetic analysis based on the nucleotide sequences of 14 protein-coding genes from the mitogenome (*atp*1, *atp*4, *atp*6, *atp*9, *ccm*B, *ccm*C, *co*b, *mat*R, *nad*3, *nad*4L, *nad*6, *nad*9, *rps*12, *rps*4). The sequence obtained from this study was highlighted in Bold. Phylogenetic analysis was conducted with the best evolutionary model “TVM + F + I + G4” and “GTR + F + G4” based on Bayesian Information Criterion (BIC) scores for the cpgenomes and mitogenomes, respectively

To study the evolution of the organelle genomes of *S. divaricata,* we conducted a phylogenetic analysis of the organelle genomes of *S. divaricata* and 10 related species. Two *Solanum* species were selected as the outgroups. In total, we used the nucleotide sequences of 71 common genes (*atp*A, *atp*B, *atp*E, *atp*F, *atp*H, *ccs*A, *cem*A, *mat*K, *ndh*A, *ndh*B, *ndh*C, *ndh*D, *ndh*E, *ndh*F, n*dh*G, *ndh*H, *ndh*I, *ndh*J, *ndh*K, *pet*A, *pet*D, *pet*G, *pet*L, *pet*N, *psa*A, *psa*B, *psa*C, *psa*I, *psa*J, *psb*A, *psb*B, *psb*C, *psb*D, *psb*E, *psb*F, *psb*H, *psb*I, *psb*J, *psb*K, *psb*L, *psb*M, *psb*N, *psb*T, *rbc*L, *rpl*14, *rpl*16, *rpl*20, *rpl*22, *rpl*2, *rpl*32, *rpl*33, *rpl*36, *rpo*A, *rpo*B, *rpo*C1, *rpo*C2, *rps*11, *rps*12, *rps*14, *rps*15, *rps*16, *rps*18, *rps*19, *rps*2, *rps*3, *rps*4, *rps*7, *rps*8, *ycf*2, *ycf*3 and *ycf*4) for cpgenome-based phylogenetic analysis (Fig. 4). By contrast, we utilised 14 common genes (*atp*1, *atp*4, *atp*6, *atp*9, *ccm*B, *ccm*C, *cob*, *mat*R, *nad*3, *nad4*L, *nad*6, *nad*9, *rps*12 and *rps*4) for the mitogenome-based phylogenetic analysis. The trees built with the cpgenome and the mitogenome clustered *S. divaricata* and *D. carota* together. The overall structures of the two trees are identical (Fig. 4).

### Substitution rates of protein-coding genes

**Fig. 5:**
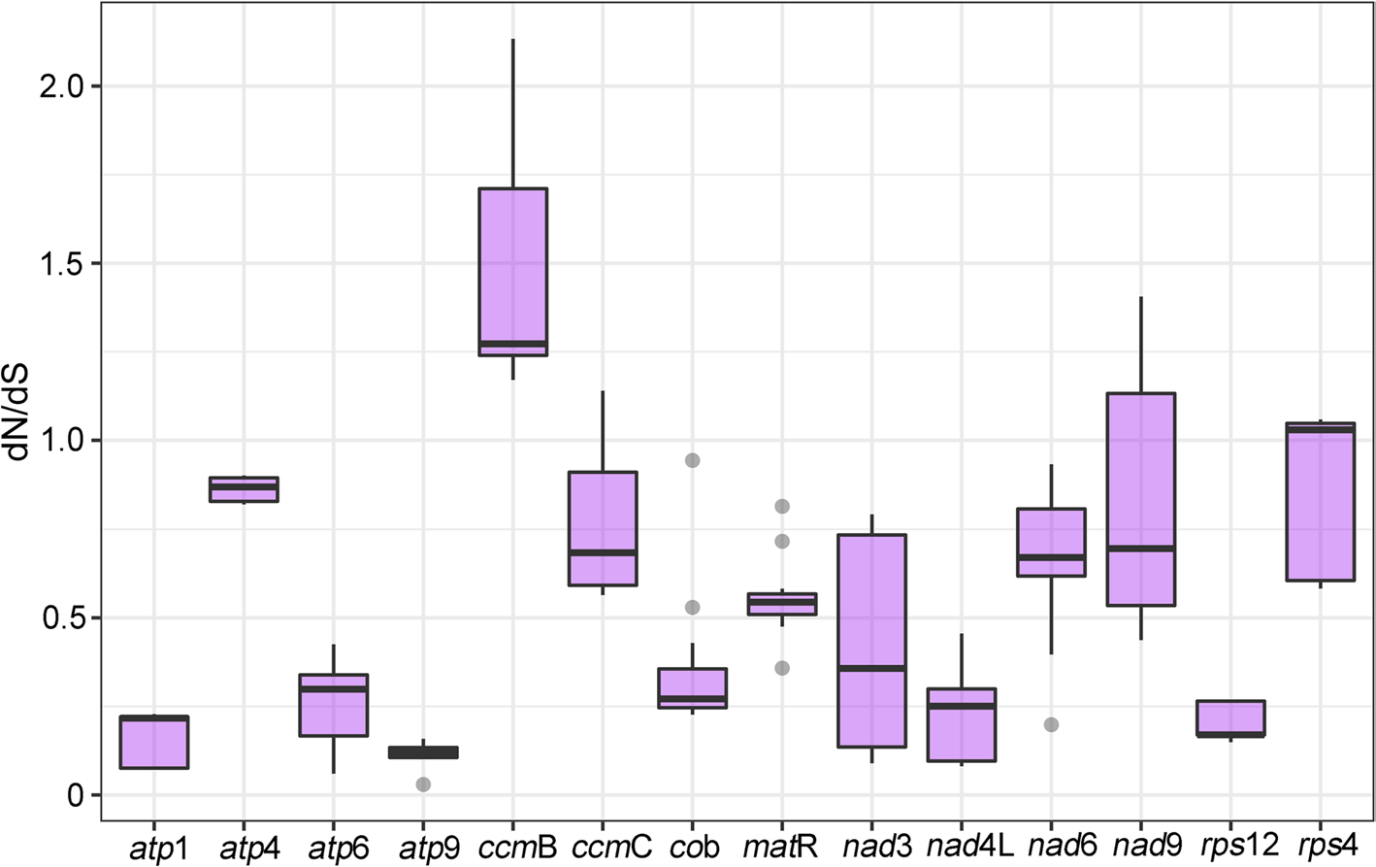
The boxplots of dN/dS values among each mitochondrial gene in the 10 related plants. The “X” axis shows the name of protein-coding genes, and the “Y” axis shows the dN/dS values

To explore the evolutionary rate of mitochondrial genes, we calculated the nonsynonymous substitution rate (dN) and the synonymous substitution rate (dS) for the 14 shared protein-coding genes. According to the criterion dN/dS > 1, there was likely a positive selection on the *ccm*B and *rps*4 genes (Fig. 5). By contrast, the other genes with a low dN/dS ratio might be under purifying selection. In particular, the *atp*9 gene has a low dN/dS radio with the smallest variations, suggesting that it is a super-conserved gene that plays a crucial role in the mitogenomes’ functioning (Table S11).

### Prediction of RNA-editing sites

**Fig. 6:**
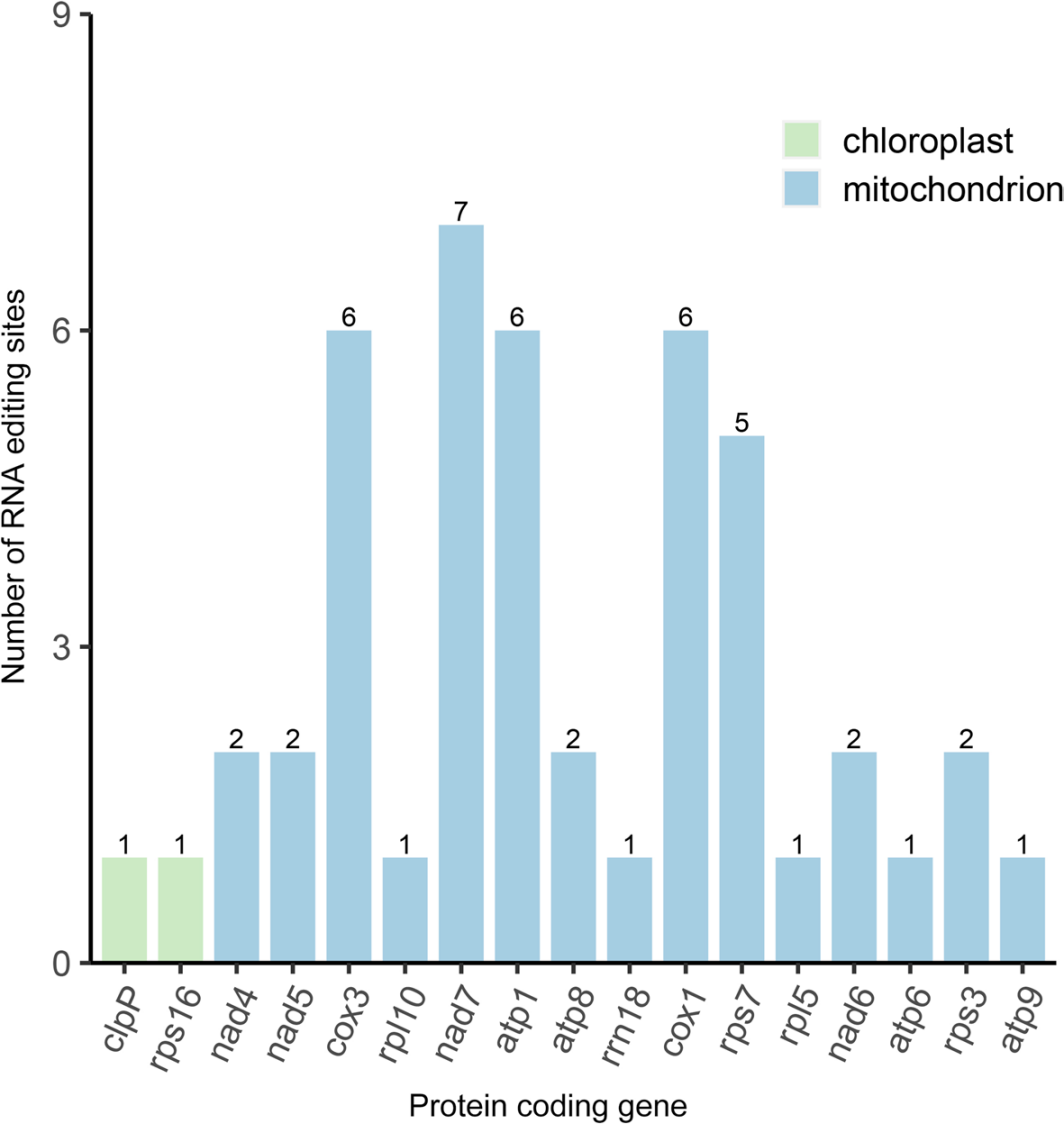
The distribution of RNA editing sites across different genes. The “X” axis shows the name of protein-coding genes, and the “Y” axis shows the number of predicted RNA editing sites

The phenomenon of RNA editing has been observed in the chloroplasts of several angiosperm plants [18]. By mapping the transcriptome data to the reference cpgenome and mitogenome, we identified 2 and 75 RNA-editing sites, respectively (Fig. 6, Table S12). The two RNA-editing sites from the cpgenome are located in the protein-coding regions of the *rps*16 and *clp*P genes. For the 75 RNA-editing sites in the mitogenome, 29 and 46 RNA-editing sites are located in the intergenic spacer regions and the protein-coding regions, respectively. These genes include the genes *nad*4, *nad*5, *nad*6, *nad*8, *nad*7, *cox*1, *cox*3, *rpl*5, *rpl*10, rps3, *rps*7, *atp*1, *atp*6, *atp*8, *atp*9 and *rrn*18. In the future, these predicted RNA-editing sites must be experimentally validated. We filtered about 27 Mb RNA reads on the basis of the organelle genomes. In total, we assembled 1220 transcripts via de novo assembly by using the Trinity software. The length of the largest transcript is 4658 bp. By comparing the protein-coding genes of the organelle genomes, we found that *rpl*5, *rps*14, *cox*1 and *rps*7 may have co-transcribed in the 150–180 kb co-linear block and had been retained during evolution (Table S13).

## Discussion

In this study, we reported the mitogenome of *S. divaricata* for the first time. The cpgenome assembled from the same sequencing data is 2 bp shorter than the published one [11]. The two indels are located in the introns of trnA-UGC (Figure S4). No similar studies have established yet whether this indel, which is in the IR region, affects the function of the genome. Comparison of the two cpgenomes using dotplot and BLASTn found no rearrangements (Figures S3–4). Phylogenomic analysis using the mitogenome and our newly assembled cpgenome showed congruent results.

**Fig. 7:**
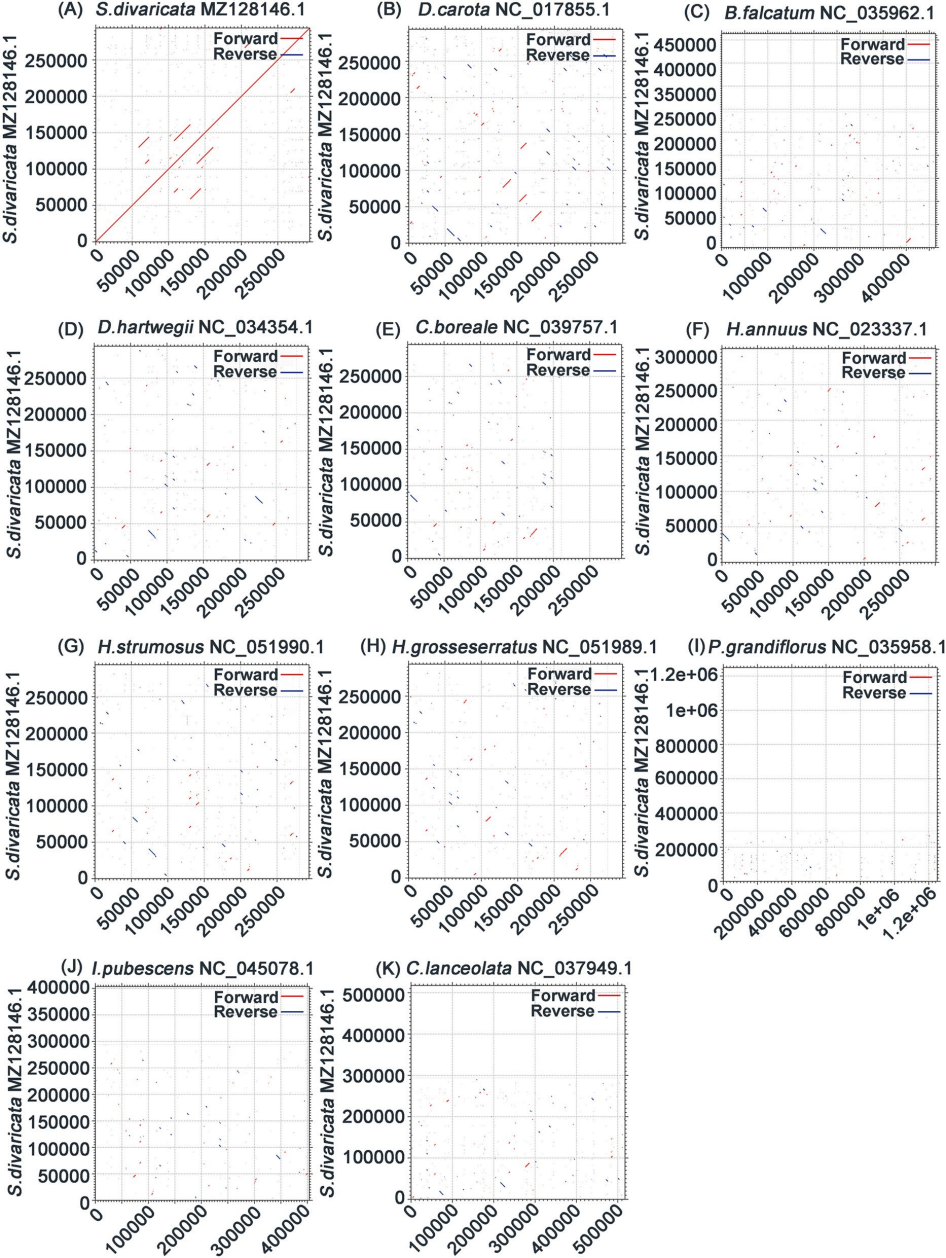
The dotplot graphs reveal collinear regions between mitogenomes in related species compared to *S. divaricata.* The red line segment represents forward direction, and the blue line segment represents reverse direction

We compared the collinearity between the published mitogenomes of related species to obtain genome rearrangement (Fig. 7). Dot plot analysis revealed that *S. divaricata* has a large number of co-linear regions with *D. carota.* The largest co-linear region is approximately 30 kb. We further predicted the possible polycistronic transcript units to determine the possible evolutionary relationships of these co-linear fragments. The results were consistent with those of the phylogenetic analysis. The co-linear blocks of the more distantly related species are small possibly because the structure of plant mitogenomes is extremely dynamic.

Comparison of the cpgenome and mitogenome sequences suggested that a DNA transfer event occurred in the cpgenome IR region (Figure S5). Mitochondrial genomes are often riddled with plastid DNA-derived sequences, called mitochondrial plastid DNAs (MTPTs) [19–21]. We counted the MTPTs in the 10 related species used in our phylogenetic analysis and obtained three conclusions. Firstly, the mitogenome of *S. divaricata* has the largest MTPT sequence (6813 bp), far exceeding the second-largest MTPT sequence in *C. lanceolata* (2995 bp) of the order Apiales (Table S14). Secondly, an MTPT of 888 bp in length is shared among the related species (Figure S5). However, the similarity (74%) is relatively low in the BLAST results. We speculate that it might represent a fragment of the cpgenome that migrated early into the mitogenome (Table S15). Lastly, this 888 bp MTPT is mostly found in the 11 Apiales species as a single-copy sequence. However, it has two copies in *P. grandiflorus* and three in *S. divaricata.*

dN/dS analysis is commonly used to identify potential selection on genes. In general, most genes in mitogenome are conserved and in neutral evolution and under purifying selection. However, two proteins, namely, *ccm*B, and *rps*4, had dN/dS ratios of > 1. Cytochrome c biogenesis protein B (*ccmB*) is a member of the ccm gene family crucial for cytochrome c biosynthesis [22]. The plant mitogenome acquired this biosynthesis process from early prokaryote cells [23, 24]. Ribosomal protein S4 (*rps*4) is one of the proteins from the small ribosomal subunit S4 that directly binds to 16S ribosomal RNA [25]. In a previous study, the *ccm*B gene was found to have undergone positive selection in Lamiales plants [26]. The biological relevance of this observation remains to be illustrated.

The ATP synthase subunit 9 (atp9) gene can be found in mitochondrial and nuclear DNA. Its migration is often a potential driving force for mitogenome evolution and is frequently used in CMS breeding [27–29]. The *atp*9 gene is strongly negatively selected in related plants, similar to those previously reported [30]. The purifying selection of the *atp*9 gene indicates that it could be used in CMS breeding of related plants.

Both mitochondria and chloroplasts had been once independent prokaryotes. Over time, cpgenomes became progressively smaller, whereas mitogenomes gradually expanded because of frequent exchanges with nuclear and chloroplast DNA [31]. In plants, the mitogenome is considerably larger than the cpgenome [30, 32, 33]. In the present study, the mitogenome (293,897 bp) is nearly twice the size of the cpgenome (147,832 bp), consistent with previous research findings. A large part of the mitogenome is similar to the cpgenome [34, 35]. Previous research has shown that MTPT regions are mutational hotspots [36]. Herein, we found 10 groups of sequences in the mitogenome of *S. divaricata*, representing 6.1% of the mitogenome, similar to cpgenome sequences. Four of these are similar to sequences in the IR regions. Thus, the sequences from the IR regions of the cpgenome can be reasonably speculated to have contributed to the expansion of the mitogenome [37].

Several efficient and accurate bioinformatics analysis software tools were used in this study to enhance the quality of the analysis results. Automatic annotation usually results in errors, such as missing 5′ and 3′ end sequences. Apollos is widely used to correct errors in automatically predicted results [38]. The standard bootstrap method is extensively used to evaluate the robustness of the phylogenetic analysis results. However, it can consume very large amounts of computing resources. UFBoot2 has improved its resampling strategies for phylogenomic data and performed better than UFBoot [39]. The REPuter software is widely employed for organelle genome repeat analysis [40]. Compared with the vmatch software, it can identify two more types of repeat sequences, namely, complement and reverse repeats. These software programs were used in this study for annotation error correction, phylogenetic analysis and repetitive sequence analysis.

Plant mitogenomes are difficult to assemble for two reasons. Firstly, there are no efficient methods for enriching plant mitochondria before DNA extraction. Secondly, unlike animal mitogenomes, plant mitogenomes are highly diverse, particularly those of angiosperms. For example, the size of plant mitogenomes ranges from 66 kb to 2 MB, making the use of a reference-based method for genome assembly challenging [41–43]. The structure of plant mitogenomes can be complex. Mitogenomes can have multiple chromosomes [44]. The presence of long repeat sequences can further complicate the assembly process. In this study, we successfully assembled the mitogenome of *S. divaricata*, thanks to the scarcity of repeat elements in the mitogenome. Nevertheless, long reads produced by third-generation sequencing technologies are needed to validate the correctness of the mitogenome [45].

## Conclusions

In this study, we reported the mitogenome of *S. divaricata* for the first time and assembled its cpgenome from the same sequencing data set. Phylogenomic analysis with the mitogenome and the cpgenome assembled showed congruent trees. We identified 10 mitochondrial DNA fragments homologous to those in the cpgenome by comparing the mitogenome and cpgenome sequences. DNA fragments from the cpgenome IR region might have transferred into the mitogenome and contributed to its length expansion. This study provides valuable information to understand the coordinated evolution of the cpgenomes and the mitogenomes of plants belonging to the family Apiaceae.

## Methods

### Plant materials, DNA extraction and sequencing

Fresh young leaves of *S. divaricata* were collected from the Institute of Medicinal Plant Development (IMPLAD), Beijing, China. Total DNA was extracted using a DNA extraction kit (Tiangen Biotech, Beijing, China) and stored at the herbarium of IMPLAD with the accession number Implad 20,170,491. DNA library was constructed from 1 μg genomic DNA, and the library was sequenced with Miseq platform (Illumina, San Diego, CA, USA).

### Genome assembly and annotation

The organelle genomes were assembled with GetOrganelle (v.1.6.4) [46]. In particular, the cpgenome was assembled with the parameters ‘-R 15 -k 21,45,65,85,105 -F embplant_pt’. By comparison, the mitogenome was assembled with the parameters ‘-R 50 -k 21,45,65,85,105 -P 1000000 -F embplant_mt’. The bandage software (v.0.8.1) tool was used to visualise the connections among contigs [47]. The cpgenome and the mitogenome were annotated using GeSeq and CPGAVAS2, respectively [48, 49]. The annotation results were manually improved by using Apollo (v.1.11.8) [38]. Lastly, the structures of the cpgenome and the mitogenome were plotted using CPGview-RSG (http://www.herbalgenomics.org/cpgview/) and OGdraw [50], respectively. The cpgenome and the mitogenome had been submitted to GenBank with the accession numbers MZ089852 and MZ128146, respectively.

### DNA transfer between the chloroplast and the mitochondrion

Sequence similarity between the cpgenome (MZ089852) and the mitogenome (MZ128146) were analysed to identify transferred DNA fragments by using BLASTN with an e-value cut-off of 1*e*-5 [51]. The results were visualised using the Circos package implemented in TBtools [52, 53].

### Analysis of repeat elements

Microsatellite sequence repeats were identified using MISA with the parameters ‘1-10 2-5 3-4 4-3 5-3 6-3’ [54]. Tandem repeats were identified using TRF with the parameters ‘2 7 7 80 10 50 500 -f -d -m’ [55]. Dispersed repeats were identified using REPuter web server (https://bibiserv.cebitec.uni-bielefeld.de/reputer/, 2001) with the parameters ‘Hamming Distance 3, Maximum Computed Repeats 5000, Minimal Repeat Size 30’ and filtered with an e-value cut-off of 1*e*-5 [40].

### Sequence alignment and phylogenetic inference

Differences in the sequences of the published cpgenome of *S. divaricata* and the cpgenomes assembled herein were compared using BLASTN with an e-value cut-off of 1*e*-5 [51]. For phylogenetic analysis, the sequences of shared genes were extracted and concatenated using Phylosuite [56]. They were then aligned using MAFFT [57]. Gblocks was utilised to select the optimal multiple sequence alignment regions with default parameters [58]. Both the cpgenome and the mitogenome of *S. divaricata* and 10 related species were subjected to phylogenetic analysis by using IQTREE [59]. Two *Solanum* species were selected as the outgroups. Phylogenetic analysis was conducted with the best evolutionary model ‘TVM + F + I + G4’ and ‘GTR + F + G4’ based on Bayesian Information Criterion scores for the cpgenomes and the mitogenomes, respectively. Bootstrap analysis was performed with 1000 replicates by using UFBoot2 (v 1.6.12) [39]. The newick format tree was visualised using iTOL6 (https://itol.embl.de/) [60].

### Selective pressure analysis

The dN/dS ratios of 14 protein-coding sequences among mitogenomes from *S. divaricata* and 10 campanulids were calculated using PAML (version 4.9) [61]. The yn00 module was selected to estimate nonsynonymous substitution rate (dN) and synonymous substitution rate (dS) with the following parameters: ‘verbose = 0, icode = 0, weighting = 0, commonf3x4 = 0, ndata = 1’. A boxplot of pairwise dN/dS values was created using the R package ggplot2 [62].

### Prediction of RNA-editing sites and polycistronic transcript units

The transcriptome data (SRR11365146) of *S. divaricata* were downloaded from the SRA database (http://www.ncbi.nlm.nih.gov/sra). The raw data were mapped to the *S. divaricata* organelle genomes by using TopHat2 [63]. RNA-editing sites were calculated using REDItools with the parameters ‘coverage ≥ 5, frequency ≥ 0.1, *p*-value ≤ 0.5’ [64]. The raw data were de novo assembled by using the Trinity program [65]. The 50 longest transcripts were selected for comparison with the genes from the organelle genome to predict polycistronic transcript units.

## Supplementary Information

**Additional file 1: Table S1.** Gene compositions of the *S. divaricata* plastome. **Table S2.** The lengths of introns and exons for the splitting genes in the *S. divaricata* plastome. **Table S3.** The The lengths of introns and exons for the splitting genes in the *S. divaricata* mitogenome. **Table S4.** Microsatellite repeats in the *S. divaricata* plastome. The structure of Microsatellite repeats is presented as repeat units surrounded with parenthesis and the numbers of repeat units. **Table S5.** Microsatellite repeats in the *S. divaricata* mitogenome. The structure of Microsatellite repeats is presented as repeat units surrounded with parenthesis and the numbers of repeat units. **Table S6.** Tandem repeats in the *S. divaricata* plastome. **Table S7.** Tandem repeats in the *S. divaricata* mitogenome. **Table S8.** Dispersed repeats in the *S. divaricata* plastome. **Table S9.** Dispersed repeats in the *S. divaricata* mitogenome. **Table S10.** DNA transfer of *S. divaricata* organelle genomes. **Table S11.** The dN, dS of the common genes of *S. divaricata* mitogenome. **Table S12.** RNA editing sites of *S. divaricata* organelle genomes. **Table S13.** PTUs identified in organelle genomes of *S. divaricata*. **Table S14.** The MTPT fragments in the Apiales species. **Table S15.** The common MTPT DNA fragments in Apiales species. **Supplementary Figure 1.** Cis-splicing gene map generated for the chloroplast genome of *S. divaricata*. **Supplementary Figure 2.** Trans-splicing gene map generated for the chloroplast genome of A. thaliana. **Supplementary Figure 3.** The dotplot of two *S. divaricata* chloroplast genomes. **Supplementary Figure 4.** The difference between the two chloroplast genomes identified by BLASTN. **Supplementary Figure 5.** Comparison of the cpgenome and mitogenome sequences suggest the transferring of DNA fragments from the cpgenome to the mitogenome.

## Data Availability

The cpgenome and mitogenome sequences supporting the conclusions of this article are available in GenBank (https://www.ncbi.nlm.nih.gov/) with accession numbers: MZ089852 and MZ128146, respectively. The sample has been deposited in the Institute of Medicinal Plant Development (Beijing, China) with their accession numbers 20170491. The raw data has been submitted to the SRA database (BioSample: SAMN20926830; BioProject: PRJNA756825; SRA: SRR15563639).
