# Supplementary material for: Comparative analysis of the chloroplast and mitochondrial genomes of Saposhnikovia divaricata revealed the possible transfer of plastome repeat regions into the mitogenome
Source: BMC Genomics. 2022 Aug 10;23:570. doi: 10.1186/s12864-022-08821-0 (PMC9364500; doi:10.1186/s12864-022-08821-0)
Supplement: Supplementary file 1 — Additional file 1: Table S1. Gene compositions of the S. divaricata plastome. Table S2. The lengths of introns and exons for the splitting genes in the S. divaricata plastome. Table S3. The The lengths of introns and exons for the splitting genes in the S. divaricata mitogenome. Table S4. Microsatellite repeats in the S. divaricata plastome. The structure of Microsatellite repeats is presented as repeat units surrounded with parenthesis and the numbers of repeat units. Table S5. Microsatellite repeats in the S. divaricata mitogenome. The structure of Microsatellite repeats is presented as repeat units surrounded with parenthesis and the numbers of repeat units. Table S6. Tandem repeats in the S. divaricata plastome. Table S7. Tandem repeats in the S. divaricata mitogenome. Table S8. Dispersed repeats in the S. divaricata plastome. Table S9. Dispersed repeats in the S. divaricata mitogenome. Table S10. DNA transfer of S. divaricata organelle genomes. Table S11. The dN, dS of the common genes of S. divaricata mitogenome. Table S12. RNA editing sites of S. divaricata organelle genomes. Table S13. PTUs identified in organelle genomes of S. divaricata. Table S14. The MTPT fragments in the Apiales species. Table S15. The common MTPT DNA fragments in Apiales species. Supplementary Figure 1. Cis-splicing gene map generated for the chloroplast genome of S. divaricata. Supplementary Figure 2. Trans-splicing gene map generated for the chloroplast genome of A. thaliana. Supplementary Figure 3. The dotplot of two S. divaricata chloroplast genomes. Supplementary Figure 4. The difference between the two chloroplast genomes identified by BLASTN. Supplementary Figure 5. Comparison of the cpgenome and mitogenome sequences suggest the transferring of DNA fragments from the cpgenome to the mitogenome. [file 12864_2022_8821_MOESM1_ESM.docx]

**Table and figure legend:**

# Table S1 Gene compositions of the *S. divaricata* plastome.

| Category of genes | Group of genes | Name of genes |
| --- | --- | --- |
|  | rRNA | *rrn*4.5S (×2), *rrn*5S (×2), *rrn*16S (×2), *rrn*23S (×2) |
|  | tRNA | *trn*A-UGC (×2)*, trn*C-GCA, *trn*D-GUC*, trn*E-UUC*,*  *trn*F-GAA*, trn*fM-CAU, *trn*G-GCC*, trn*G-UCC*,*  *trn*H-GUG, *trn*I-CAU*, trn*I-GAU (x2)*, trn*K-UUU,  *trn*L-CAA (x2)*, trn*L-UAA*, trn*L-UAG*, trn*M-CAU*,*  *trn*N-GUU (x2), *trn*P-UGG*, trn*Q-UUG*, trn*R-UCU*,*  *trn*R-ACG (x2), *trn*S-GCU*, trn*S-GGA, *trn*S-UGA*,*  *trn*T-GGU*, trn*T-UGU*, trn*V-GAC (x2), *trn*V-UAC,  *trn*W-CCA, *trn*Y-GUA |
| photosynthesis | Subunits of ATP synthase  Subunits of photosystem II | *atp*A*, atp*B*, atp*E*, atp*F*, atp*H*, atp*I  *psb*A*, psb*B*, psb*C*, psb*D*, psb*E, *psb*F*, psb*H*, psb*I, *psb*J, *psb*K, *psb*L, *psb*M, *psb*N, *psb*T, *psb*Z, *ycf*3 |
|  | Subunits of cytochrome b/f complex | *pet*A*, pet*B*, pet*D*, pet*G*, pet*L, *pet*N |
|  | Subunits of photosystem I | *psa*A*, psa*B*, psa*C*, psa*I*, psa*J |
|  | Subunit of rubisco | *rbc*L |
|  | Subunits of NADH-dehydrogenase | *ndh*A*, ndh*B (×2)*, ndh*C*, ndh*D*, ndh*E*, ndh*F*, ndh*G*, ndh*H*, ndh*I*, ndh*J, *ndh*K |
| Self-replication  Other genes | Large subunit of ribosome | *rpl*14*, rpl*16*, rpl*2*, rpl*20*, rpl*22*, rpl*23*, rpl*32*, rpl*33*, rpl*36 |
|  | DNA dependent RNA polymerase | *rpo*A, *rpo*B, *rpo*C1, *rpo*C2 |
|  | Small subunit of ribosome | *rps*11*, rps*12 (×2)*, rps*14*, rps*15*, rps*16*, rps*18*, rps*19*, rps*2*, rps*3, *rps*4, *rps*7 (×2)*, rps*8 |
|  | Subunit of Acetyl-CoA-carboxylase | *acc*D |
|  | c-type cytochrom synthesis gene | *ccs*A |
|  | Envelop membrane protein | *cem*A |
|  | Protease | *clp*P |
|  | Translational initiation | *inf*A |
|  | Maturase | *mat*K |
| Unknown | Conserves open reading frames | *ycf*1 (×2)*, ycf*2*, ycf*4*, ycf*15 (×2) |

# Table S2 The lengths of introns and exons for the splitting genes in the *S. divaricata* plastome.

| Gene | Strand | Start | End | Exon I | Intron I | Exon II | Intron II | Exon III |
| --- | --- | --- | --- | --- | --- | --- | --- | --- |
| *trn*K-UUU | - | 1578 | 4181 | 37 | 2532 | 35 |  |  |
| *rps*16 | - | 4902 | 5997 | 40 | 859 | 197 |  |  |
| *trn*G-UCC | + | 9048 | 9821 | 23 | 703 | 48 |  |  |
| *atp*F | - | 11731 | 12987 | 145 | 711 | 401 |  |  |
| *rpo*C1 | - | 21002 | 23807 | 453 | 748 | 1605 |  |  |
| *ycf*3 | - | 43485 | 45484 | 124 | 717 | 230 | 776 | 153 |
| *trn*L-UAA | + | 48507 | 49093 | 35 | 502 | 50 |  |  |
| *trn*V-UAC | - | 52713 | 53355 | 39 | 569 | 35 |  |  |
| *clp*P | - | 70485 | 72561 | 71 | 848 | 294 | 635 | 229 |
| *pet*B | + | 75522 | 76927 | 9 | 755 | 642 |  |  |
| *pet*D | + | 77109 | 78341 | 8 | 750 | 475 |  |  |
| *rpl*16 | - | 81825 | 83182 | 9 | 950 | 399 |  |  |
| *rpl*2 | - | 84892 | 86370 | 394 | 651 | 434 |  |  |
| *ndh*B | - | 95094 | 97308 | 775 | 682 | 758 |  |  |
| *trn*I-GAU | + | 102730 | 103769 | 37 | 968 | 35 |  |  |
| *trn*A-UGC | + | 103834 | 104723 | 38 | 817 | 35 |  |  |
| *ndh*A | - | 121904 | 124094 | 553 | 1099 | 539 |  |  |
| *trn*A-UGC | - | 136312 | 137201 | 38 | 817 | 35 |  |  |
| *trn*I-GAU | - | 137266 | 138305 | 37 | 968 | 35 |  |  |
| *ndh*B | + | 143727 | 145941 | 775 | 682 | 758 |  |  |

**Table S3 The The lengths of introns and exons for the splitting genes in the *S. divaricata* mitogenome.**

| Gene | Strand | Start | End | Length (bp) | | | | | | |  |  |
| --- | --- | --- | --- | --- | --- | --- | --- | --- | --- | --- | --- | --- |
|  |  |  |  | Exon I | Intron I | Exon II | Intron II | Exon III | Intron III | Exon IV | Intron IV | Exon V |
| *nad*1 | *+* | 135,529 | 243,602 | 379 | 105,857 | 88 | 1,456 | 298 |  |  |  |  |
| *nad*2 | *+* | 36,153 | 40,889 | 153 | 2,319 | 574 | 1,507 | 188 | 2,769 | 91 |  |  |
| *nad*4 | *+* | 10,972 | 19,811 | 461 | 1,407 | 516 | 3,182 | 420 | 64,480 | 394 | 1,193 | 151 |
| *nad*5 | *+* | 48,024 | 259,313 | 227 | 847 | 1,220 | 142,765 | 21 | 981 | 246 | 1,805 | 261 |
| *nad*7 | *-* | 84,222 | 78,021 | 143 | 902 | 69 | 1,337 | 466 |  |  |  |  |
| *ccm*Fc | *-* | 6,494 | 4,397 | 769 | 777 | 554 |  |  |  |  |  |  |
| *cox*2 | *+* | 129,919 | 133,148 | 386 | 1,372 | 310 | 1,082 | 84 |  |  |  |  |
| *rps*3 | *-* | 267,107 | 263,511 | 74 | 1,766 | 1,759 |  |  |  |  |  |  |

**Table S4 Microsatellite repeats in the *S. divaricata* plastome. The structure of Microsatellite repeats is presented as repeat units surrounded with parenthesis and the numbers of repeat units.**

| Type | Structure of Microsatellite Repeats | Size (bp) | | Start | End |
| --- | --- | --- | --- | --- | --- |
| p4 | (TTTA)4 | 16 | 145 | | 160 |
| p1 | (A)10 | 10 | 1539 | | 1548 |
| p1 | (A)10 | 10 | 1794 | | 1803 |
| p4 | (AATA)3 | 12 | 3871 | | 3882 |
| p2 | (TA)5 | 10 | 4811 | | 4820 |
| p3 | (TTA)5 | 15 | 5419 | | 5433 |
| p1 | (A)10 | 10 | 9393 | | 9402 |
| p2 | (AT)7 | 14 | 9867 | | 9880 |
| p2 | (AT)5 | 10 | 10059 | | 10068 |
| p2 | (AT)9 | 18 | 13059 | | 13076 |
| p4 | (TTTA)4 | 16 | 13138 | | 13153 |
| p1 | (A)14 | 14 | 16406 | | 16419 |
| p1 | (T)11 | 11 | 18651 | | 18661 |
| p2 | (AT)5 | 10 | 20015 | | 20024 |
| p4 | (AAAT)3 | 12 | 22840 | | 22851 |
| p1 | (T)12 | 12 | 26380 | | 26391 |
| p1 | (A)10 | 10 | 27392 | | 27401 |
| p3 | (AAT)6 | 18 | 28642 | | 28659 |
| p1 | (T)12 | 12 | 29602 | | 29613 |
| p2 | (AT)5 | 10 | 30094 | | 30103 |
| p5 | (TATAT)3 | 15 | 32165 | | 32179 |
| p1 | (T)12 | 12 | 32753 | | 32764 |
| p1 | (A)12 | 12 | 33320 | | 33331 |
| p1 | (C)10 | 10 | 37141 | | 37150 |
| p4 | (ATTT)3 | 12 | 42904 | | 42915 |
| p1 | (A)13 | 13 | 43455 | | 43467 |
| p1 | (T)10 | 10 | 45269 | | 45278 |
| p2 | (TA)7 | 14 | 47465 | | 47478 |
| p2 | (AT)5 | 10 | 50265 | | 50274 |
| p2 | (TA)5 | 10 | 51913 | | 51922 |
| p2 | (TA)7 | 14 | 51926 | | 51939 |
| p1 | (T)10 | 10 | 52688 | | 52697 |
| p1 | (T)10 | 10 | 55648 | | 55657 |
| p3 | (ATA)4 | 12 | 55702 | | 55713 |
| p1 | (A)18 | 18 | 56234 | | 56251 |
| p1 | (T)10 | 10 | 58021 | | 58030 |
| p1 | (T)10 | 10 | 60531 | | 60540 |
| p1 | (A)10 | 10 | 64123 | | 64132 |
| p2 | (TA)6 | 12 | 64145 | | 64156 |
| p2 | (TA)5 | 10 | 64158 | | 64167 |
| p4 | (TCCT)3 | 12 | 68077 | | 68088 |
| p1 | (A)11 | 11 | 71813 | | 71823 |
| p1 | (A)10 | 10 | 71889 | | 71898 |
| p1 | (T)10 | 10 | 72637 | | 72646 |
| p2 | (AT)5 | 10 | 77722 | | 77731 |
| p1 | (T)12 | 12 | 83124 | | 83135 |
| p2 | (AT)5 | 10 | 84523 | | 84532 |
| p1 | (T)16 | 16 | 84843 | | 84858 |
| p1 | (G)13 | 13 | 94287 | | 94299 |
| p1 | (T)13 | 13 | 99335 | | 99347 |
| p1 | (T)10 | 10 | 103203 | | 103212 |
| p1 | (G)13 | 13 | 104444 | | 104456 |
| p4 | (AGGT)3 | 12 | 106534 | | 106545 |
| p1 | (A)10 | 10 | 111048 | | 111057 |
| p1 | (A)11 | 11 | 111832 | | 111842 |
| p1 | (A)12 | 12 | 115538 | | 115549 |
| p2 | (TA)6 | 12 | 115668 | | 115679 |
| p2 | (TA)8 | 16 | 115683 | | 115698 |
| p2 | (AT)6 | 12 | 115713 | | 115724 |
| p4 | (TCTT)3 | 12 | 116728 | | 116739 |
| p1 | (A)13 | 13 | 116775 | | 116787 |
| p1 | (A)11 | 11 | 120332 | | 120342 |
| p2 | (TA)5 | 10 | 120940 | | 120949 |
| p1 | (T)10 | 10 | 121129 | | 121138 |
| p4 | (CAAT)3 | 12 | 121202 | | 121213 |
| p2 | (TA)5 | 10 | 127518 | | 127527 |
| p1 | (T)15 | 15 | 128045 | | 128059 |
| p1 | (T)11 | 11 | 128409 | | 128419 |
| p1 | (T)10 | 10 | 128670 | | 128679 |
| p1 | (T)11 | 11 | 129193 | | 129203 |
| p1 | (T)10 | 10 | 129978 | | 129987 |
| p4 | (CTAC)3 | 12 | 134488 | | 134499 |
| p1 | (C)13 | 13 | 136579 | | 136591 |
| p1 | (A)10 | 10 | 137823 | | 137832 |
| p1 | (A)13 | 13 | 141688 | | 141700 |
| p1 | (C)13 | 13 | 146736 | | 146748 |

# Table S5 Microsatellite repeats in the *S. divaricata* mitogenome*.* The structure of Microsatellite repeats is presented as repeat units surrounded with parenthesis and the numbers of repeat units.

| Type | Structure of Microsatellite Repeats | Size (bp) | | Start | End |
| --- | --- | --- | --- | --- | --- |
| p1 | (C)13 | 13 | 117108 | | 117120 |
| p1 | (A)10 | 10 | 118352 | | 118361 |
| p3 | (GCC)4 | 12 | 120436 | | 120447 |
| p1 | (A)11 | 11 | 120871 | | 120881 |
| p4 | (ACTA)3 | 12 | 124951 | | 124962 |
| p1 | (T)10 | 10 | 127202 | | 127211 |
| p5 | (AGATG)3 | 15 | 134119 | | 134133 |
| p3 | (ATA)5 | 15 | 136175 | | 136189 |
| p3 | (GCT)4 | 12 | 138541 | | 138552 |
| p4 | (GGCG)3 | 12 | 142870 | | 142881 |
| p4 | (CTAC)3 | 12 | 146333 | | 146344 |
| p1 | (C)13 | 13 | 148424 | | 148436 |
| p1 | (A)10 | 10 | 149668 | | 149677 |
| p3 | (GCC)4 | 12 | 151752 | | 151763 |
| p1 | (A)11 | 11 | 152187 | | 152197 |
| p4 | (ACTA)3 | 12 | 156267 | | 156278 |
| p1 | (T)10 | 10 | 158518 | | 158527 |
| p3 | (CTT)4 | 12 | 167081 | | 167092 |
| p5 | (CTATA)4 | 20 | 178612 | | 178631 |
| p4 | (CTTT)3 | 12 | 179675 | | 179686 |
| p2 | (AT)7 | 14 | 180247 | | 180260 |
| p4 | (AGCA)3 | 12 | 188085 | | 188096 |
| p1 | (T)12 | 12 | 191943 | | 191954 |
| p4 | (AAAG)3 | 12 | 202416 | | 202427 |
| p4 | (TTCT)3 | 12 | 208224 | | 208235 |
| p4 | (AAAG)3 | 12 | 218096 | | 218107 |
| p4 | (ATTC)3 | 12 | 218601 | | 218612 |
| p1 | (A)10 | 10 | 226248 | | 226257 |
| p2 | (AT)5 | 10 | 249219 | | 249228 |
| p3 | (CTT)4 | 12 | 252828 | | 252839 |
| p4 | (AAGC)3 | 12 | 259353 | | 259364 |
| p5 | (CCATA)3 | 15 | 262462 | | 262476 |
| p1 | (T)12 | 12 | 263676 | | 263687 |
| p4 | (AATG)3 | 12 | 265127 | | 265138 |
| p2 | (AC)5 | 10 | 265724 | | 265733 |
| p1 | (T)12 | 12 | 266966 | | 266977 |
| p4 | (TTCT)3 | 12 | 270890 | | 270901 |
| p4 | (TTCT)3 | 12 | 274320 | | 274331 |
| p2 | (AG)5 | 10 | 282086 | | 282095 |
| p4 | (AAAG)3 | 12 | 292178 | | 292189 |
| p4 | (AAAG)3 | 12 | 292216 | | 292227 |

# Table S6 Tandem repeats in the *S. divaricata* plastome.

| Start and End Sites of the Tandem Repeats | Period  Size（bp） | Copy Number | Consensus Size  （bp） | Percent Matches | Indels | Score | Bases number  A G C T | | | | Extropy （0-2） |
| --- | --- | --- | --- | --- | --- | --- | --- | --- | --- | --- | --- |
| 4601-4631 | 13 | 2.4 | 13 | 100 | 0 | 62 | 58 | 6 | 12 | 22 | 1.58 |
| 7111-7147 | 16 | 2.3 | 16 | 100 | 0 | 74 | 54 | 5 | 13 | 27 | 1.61 |
| 9845-9879 | 15 | 2.4 | 15 | 95 | 4 | 63 | 54 | 5 | 0 | 40 | 1.24 |
| 31758-31787 | 15 | 2 | 15 | 100 | 0 | 60 | 53 | 0 | 6 | 40 | 1.27 |
| 32932-32969 | 18 | 2.1 | 18 | 100 | 0 | 76 | 44 | 5 | 10 | 39 | 1.61 |
| 36750-36787 | 19 | 2 | 19 | 100 | 0 | 76 | 36 | 0 | 10 | 52 | 1.36 |
| 51908-51955 | 17 | 2.9 | 17 | 93 | 6 | 82 | 39 | 0 | 0 | 60 | 0.97 |
| 67923-67971 | 24 | 2 | 24 | 100 | 0 | 98 | 44 | 8 | 12 | 34 | 1.71 |
| 68403-68451 | 24 | 2 | 24 | 92 | 0 | 80 | 46 | 8 | 4 | 40 | 1.52 |
| 74827-74863 | 19 | 1.9 | 19 | 94 | 0 | 65 | 54 | 13 | 8 | 24 | 1.66 |
| 83214-83255 | 20 | 2.1 | 20 | 100 | 0 | 84 | 33 | 7 | 14 | 45 | 1.72 |
| 90174-90217 | 15 | 2.9 | 15 | 93 | 0 | 70 | 27 | 34 | 13 | 25 | 1.93 |
| 91434-91535 | 18 | 5.7 | 18 | 98 | 0 | 186 | 28 | 8 | 27 | 35 | 1.87 |
| 94004-94068 | 21 | 3.1 | 21 | 97 | 0 | 121 | 41 | 4 | 36 | 16 | 1.7 |
| 99906-99968 | 21 | 3 | 21 | 100 | 0 | 126 | 14 | 23 | 14 | 47 | 1.8 |
| 103274-103314 | 20 | 2 | 20 | 100 | 0 | 82 | 51 | 14 | 29 | 4 | 1.63 |
| 107943-108008 | 32 | 2.1 | 32 | 97 | 0 | 123 | 40 | 24 | 9 | 25 | 1.84 |
| 111867-111909 | 17 | 2.5 | 17 | 96 | 3 | 77 | 51 | 0 | 0 | 48 | 1 |
| 115663-115697 | 17 | 2.1 | 17 | 94 | 0 | 61 | 37 | 2 | 0 | 60 | 1.12 |
| 119672-119707 | 18 | 2 | 18 | 100 | 0 | 72 | 38 | 16 | 0 | 44 | 1.48 |
| 122640-122671 | 15 | 2.1 | 15 | 100 | 0 | 64 | 28 | 0 | 0 | 71 | 0.86 |
| 133027-133092 | 32 | 2.1 | 32 | 97 | 0 | 123 | 25 | 9 | 24 | 40 | 1.84 |
| 137721-137761 | 20 | 2 | 20 | 100 | 0 | 82 | 4 | 29 | 14 | 51 | 1.63 |
| 141067-141129 | 21 | 3 | 21 | 100 | 0 | 126 | 47 | 14 | 23 | 14 | 1.8 |
| 146967-147031 | 21 | 3.1 | 21 | 97 | 0 | 121 | 16 | 36 | 4 | 41 | 1.7 |

Parameters: Total length (Period size * Copy number) >= 30 and Percent Matches >= 90%

# Table S7 Tandem repeats in the *S. divaricata* mitogenome.

| Start and End Sites of the Tandem Repeats | Period  Size（bp） | Copy Number | Consensus Size  （bp） | Percent Matches | Indels | Score | Bases number  A G C T | | | | Extropy （0-2） |
| --- | --- | --- | --- | --- | --- | --- | --- | --- | --- | --- | --- |
| 30226-30255 | 12 | 2.6 | 12 | 94 | 5 | 53 | 53 | 0 | 13 | 33 | 1.4 |
| 67850-67926 | 12 | 6.5 | 12 | 87 | 3 | 102 | 9 | 55 | 0 | 35 | 1.31 |
| 67860-67926 | 35 | 1.9 | 35 | 93 | 0 | 116 | 10 | 52 | 0 | 37 | 1.36 |
| 89226-89261 | 7 | 5.1 | 7 | 100 | 0 | 72 | 41 | 0 | 30 | 27 | 1.56 |
| 99556-99592 | 9 | 4.1 | 9 | 100 | 0 | 74 | 32 | 21 | 0 | 45 | 1.52 |
| 113222-113249 | 14 | 2 | 14 | 100 | 0 | 56 | 14 | 7 | 0 | 78 | 0.95 |
| 113556-113621 | 32 | 2.1 | 32 | 97 | 0 | 123 | 25 | 9 | 24 | 40 | 1.84 |
| 118250-118290 | 20 | 2 | 20 | 100 | 0 | 82 | 4 | 29 | 14 | 51 | 1.63 |
| 138769-138845 | 12 | 6.5 | 12 | 87 | 3 | 102 | 9 | 55 | 0 | 35 | 1.31 |
| 138779-138845 | 35 | 1.9 | 35 | 93 | 0 | 116 | 10 | 52 | 0 | 37 | 1.36 |
| 144538-144565 | 14 | 2 | 14 | 100 | 0 | 56 | 14 | 7 | 0 | 78 | 0.95 |
| 144872-144937 | 32 | 2.1 | 32 | 97 | 0 | 123 | 25 | 9 | 24 | 40 | 1.84 |
| 149566-149606 | 20 | 2 | 20 | 100 | 0 | 82 | 4 | 29 | 14 | 51 | 1.63 |
| 169008-169042 | 16 | 2.2 | 16 | 100 | 0 | 70 | 37 | 17 | 28 | 17 | 1.92 |
| 171140-171172 | 17 | 1.9 | 17 | 100 | 0 | 66 | 51 | 6 | 12 | 30 | 1.63 |
| 188359-188418 | 10 | 6 | 10 | 100 | 0 | 120 | 60 | 20 | 10 | 10 | 1.57 |
| 192342-192373 | 15 | 2.1 | 15 | 100 | 0 | 64 | 34 | 12 | 18 | 34 | 1.89 |
| 193939-193974 | 16 | 2.2 | 16 | 90 | 0 | 54 | 55 | 30 | 8 | 5 | 1.52 |
| 205908-205939 | 15 | 2.1 | 15 | 94 | 0 | 55 | 9 | 31 | 12 | 46 | 1.73 |
| 239945-240081 | 71 | 1.9 | 71 | 91 | 4 | 224 | 35 | 13 | 27 | 22 | 1.92 |
| 268574-268605 | 15 | 2.1 | 15 | 94 | 0 | 55 | 9 | 31 | 12 | 46 | 1.73 |
| 275406-275454 | 24 | 2 | 24 | 96 | 0 | 89 | 38 | 24 | 22 | 14 | 1.91 |
| 283840-283906 | 25 | 2.7 | 25 | 90 | 0 | 116 | 40 | 16 | 26 | 16 | 1.89 |
| 286391-286434 | 15 | 2.9 | 15 | 100 | 0 | 88 | 47 | 13 | 18 | 20 | 1.82 |
| 288657-288746 | 27 | 3.3 | 27 | 93 | 0 | 144 | 32 | 21 | 18 | 27 | 1.97 |
| 293108-293143 | 7 | 5.1 | 7 | 100 | 0 | 72 | 41 | 0 | 30 | 27 | 1.56 |

# Table S8 Dispersed repeats in the *S. divaricata* plastome.

| The repeat length of the first part | The starting site of the first part | Matching direction | The repeat length of the second part | The starting site of the second part | interval distance of repeats | E-value |
| --- | --- | --- | --- | --- | --- | --- |
| 84 | 91433 | F | 84 | 91451 | -1 | 4.14E-39 |
| 70 | 91433 | F | 70 | 91469 | -3 | 6.52E-27 |
| 59 | 91458 | F | 59 | 91476 | 0 | 1.85E-26 |
| 52 | 91433 | F | 52 | 91487 | -3 | 1.81E-16 |
| 42 | 99905 | F | 42 | 99926 | 0 | 3.18E-16 |
| 42 | 99905 | P | 42 | 141066 | 0 | 3.18E-16 |
| 42 | 99926 | P | 42 | 141087 | 0 | 3.18E-16 |
| 42 | 141066 | F | 42 | 141087 | 0 | 3.18E-16 |
| 41 | 91458 | F | 41 | 91494 | 0 | 1.27E-15 |
| 44 | 94003 | F | 44 | 94024 | -1 | 2.62E-15 |
| 44 | 94003 | P | 44 | 146966 | -1 | 2.62E-15 |
| 44 | 94024 | P | 44 | 146987 | -1 | 2.62E-15 |
| 44 | 146966 | F | 44 | 146987 | -1 | 2.62E-15 |
| 36 | 94011 | F | 36 | 94032 | 0 | 1.30E-12 |
| 36 | 94011 | P | 36 | 146966 | 0 | 1.30E-12 |
| 36 | 94032 | P | 36 | 146987 | 0 | 1.30E-12 |
| 45 | 74889 | P | 45 | 74889 | -3 | 1.90E-12 |
| 39 | 31021 | P | 39 | 31550 | -1 | 2.38E-12 |
| 40 | 115670 | P | 40 | 115670 | -2 | 3.57E-11 |
| 33 | 51916 | R | 33 | 51916 | 0 | 8.33E-11 |
| 39 | 44679 | F | 39 | 98962 | -2 | 1.36E-10 |
| 39 | 44679 | P | 39 | 142033 | -2 | 1.36E-10 |
| 32 | 30082 | P | 32 | 30082 | 0 | 3.33E-10 |
| 41 | 98960 | F | 41 | 122480 | -3 | 3.66E-10 |
| 41 | 122480 | P | 41 | 142033 | -3 | 3.66E-10 |
| 38 | 111872 | P | 38 | 111872 | -2 | 5.15E-10 |
| 31 | 122639 | R | 31 | 122639 | 0 | 1.33E-09 |
| 34 | 107942 | F | 34 | 107974 | -1 | 2.12E-09 |
| 34 | 107942 | P | 34 | 133026 | -1 | 2.12E-09 |
| 34 | 107974 | P | 34 | 133058 | -1 | 2.12E-09 |
| 34 | 133026 | F | 34 | 133058 | -1 | 2.12E-09 |
| 39 | 44679 | F | 39 | 122482 | -3 | 5.02E-09 |
| 30 | 8405 | P | 30 | 46356 | 0 | 5.33E-09 |
| 31 | 51905 | R | 31 | 64144 | -1 | 1.24E-07 |
| 31 | 51912 | R | 31 | 115662 | -1 | 1.24E-07 |
| 33 | 98968 | F | 33 | 122488 | -2 | 3.96E-07 |
| 33 | 122488 | P | 33 | 142033 | -2 | 3.96E-07 |
| 30 | 115665 | R | 30 | 115667 | -1 | 4.80E-07 |
| 35 | 20788 | F | 35 | 20837 | -3 | 9.20E-07 |
| 35 | 44682 | F | 35 | 95893 | -3 | 9.20E-07 |
| 35 | 44682 | P | 35 | 145106 | -3 | 9.20E-07 |
| 32 | 52673 | R | 32 | 52673 | -2 | 1.49E-06 |
| 34 | 91433 | F | 34 | 91505 | -3 | 3.36E-06 |
| 31 | 65587 | P | 31 | 65620 | -2 | 5.58E-06 |
| 30 | 8402 | F | 30 | 36452 | -2 | 2.09E-05 |
| 30 | 51912 | F | 30 | 115669 | -2 | 2.09E-05 |
| 30 | 115667 | R | 30 | 115667 | -2 | 2.09E-05 |
| 31 | 51912 | R | 31 | 115664 | -3 | 1.62E-04 |
| 31 | 67922 | F | 31 | 67946 | -3 | 1.62E-04 |
| 31 | 108295 | P | 31 | 108295 | -3 | 1.62E-04 |
| 31 | 108295 | F | 31 | 132708 | -3 | 1.62E-04 |
| 31 | 114346 | F | 31 | 114378 | -3 | 1.62E-04 |
| 31 | 115667 | P | 31 | 115667 | -3 | 1.62E-04 |
| 31 | 115667 | F | 31 | 115669 | -3 | 1.62E-04 |
| 31 | 115667 | C | 31 | 115685 | -3 | 1.62E-04 |
| 31 | 132708 | P | 31 | 132708 | -3 | 1.62E-04 |
| 30 | 9791 | F | 30 | 37443 | -3 | 5.84E-04 |
| 30 | 9862 | R | 30 | 115668 | -3 | 5.84E-04 |
| 30 | 9863 | R | 30 | 115671 | -3 | 5.84E-04 |
| 30 | 36455 | P | 30 | 46356 | -3 | 5.84E-04 |
| 30 | 39657 | F | 30 | 41881 | -3 | 5.84E-04 |
| 30 | 47457 | F | 30 | 51918 | -3 | 5.84E-04 |
| 30 | 47459 | R | 30 | 51915 | -3 | 5.84E-04 |
| 30 | 51911 | P | 30 | 115682 | -3 | 5.84E-04 |
| 30 | 51912 | F | 30 | 115667 | -3 | 5.84E-04 |
| 30 | 51912 | C | 30 | 115670 | -3 | 5.84E-04 |
| 30 | 51922 | F | 30 | 64141 | -3 | 5.84E-04 |
| 30 | 89006 | F | 30 | 89048 | -3 | 5.84E-04 |
| 30 | 90173 | F | 30 | 90188 | -3 | 5.84E-04 |
| 30 | 91458 | F | 30 | 91512 | -3 | 5.84E-04 |
| 30 | 115529 | C | 30 | 128036 | -3 | 5.84E-04 |
| 30 | 115667 | C | 30 | 115668 | -3 | 5.84E-04 |

P: palindromic repeat; F: forward repeat (direct repeat); C: complementary repeat; R: reverse repeat

# Table S9 Dispersed repeats in the *S. divaricata* mitogenome.

| The repeat length of the first part | The starting site of the first part | Matching direction | The repeat length of the second part | The starting site of the second part | interval distance of repeats | E-value |
| --- | --- | --- | --- | --- | --- | --- |
| 22397 | 107544 | F | 22397 | 138860 | 0 | 0.00E+00 |
| 14372 | 58885 | F | 14372 | 129804 | 0 | 0.00E+00 |
| 5742 | 204828 | F | 5742 | 267494 | 0 | 0.00E+00 |
| 5316 | 67941 | F | 5316 | 107544 | 0 | 0.00E+00 |
| 1638 | 88377 | F | 1638 | 292259 | -1 | 0.00E+00 |
| 1628 | 88387 | F | 1628 | 292269 | 0 | 0.00E+00 |
| 185 | 194224 | P | 185 | 263491 | -2 | 1.55E-96 |
| 168 | 194241 | P | 168 | 263491 | 0 | 1.74E-91 |
| 137 | 58885 | F | 137 | 161120 | 0 | 8.00E-73 |
| 117 | 224477 | F | 117 | 289083 | -1 | 3.09E-58 |
| 101 | 41581 | P | 101 | 101691 | 0 | 3.78E-51 |
| 104 | 21976 | F | 104 | 193754 | -1 | 1.84E-50 |
| 97 | 26889 | F | 97 | 232316 | -1 | 2.82E-46 |
| 91 | 40930 | P | 91 | 181619 | 0 | 3.96E-45 |
| 83 | 26903 | F | 83 | 232330 | 0 | 2.60E-40 |
| 78 | 178175 | F | 78 | 258348 | -2 | 7.19E-33 |
| 81 | 107434 | F | 81 | 259866 | -3 | 9.57E-33 |
| 69 | 183961 | F | 69 | 184904 | 0 | 6.97E-32 |
| 75 | 198676 | P | 75 | 281312 | -3 | 3.10E-29 |
| 71 | 107444 | F | 71 | 259876 | -2 | 9.75E-29 |
| 61 | 120820 | P | 61 | 263676 | 0 | 4.57E-27 |
| 61 | 152136 | P | 61 | 263676 | 0 | 4.57E-27 |
| 68 | 47267 | F | 68 | 265912 | -2 | 5.72E-27 |
| 62 | 27985 | F | 62 | 265974 | -1 | 2.12E-25 |
| 61 | 28417 | P | 61 | 207559 | -1 | 8.36E-25 |
| 61 | 28417 | P | 61 | 270225 | -1 | 8.36E-25 |
| 57 | 42089 | F | 57 | 69965 | 0 | 1.17E-24 |
| 57 | 42089 | F | 57 | 109568 | 0 | 1.17E-24 |
| 57 | 42089 | F | 57 | 140884 | 0 | 1.17E-24 |
| 56 | 3715 | F | 56 | 167500 | 0 | 4.68E-24 |
| 56 | 228299 | P | 56 | 243870 | 0 | 4.68E-24 |
| 59 | 178173 | F | 59 | 243841 | -1 | 1.29E-23 |
| 62 | 88887 | F | 62 | 125901 | -2 | 1.94E-23 |
| 62 | 88887 | F | 62 | 157217 | -2 | 1.94E-23 |
| 62 | 125901 | F | 62 | 292769 | -2 | 1.94E-23 |
| 62 | 157217 | F | 62 | 292769 | -2 | 1.94E-23 |
| 54 | 243846 | F | 54 | 258351 | 0 | 7.49E-23 |
| 53 | 184940 | F | 53 | 204964 | 0 | 2.99E-22 |
| 53 | 184940 | F | 53 | 267630 | 0 | 2.99E-22 |
| 62 | 48660 | P | 62 | 266447 | -3 | 1.17E-21 |
| 62 | 107484 | F | 62 | 259916 | -3 | 1.17E-21 |
| 54 | 198697 | P | 54 | 281312 | -1 | 1.21E-20 |
| 50 | 71074 | F | 50 | 119564 | 0 | 1.92E-20 |
| 50 | 71074 | F | 50 | 150880 | 0 | 1.92E-20 |
| 50 | 110677 | F | 50 | 119564 | 0 | 1.92E-20 |
| 50 | 110677 | F | 50 | 150880 | 0 | 1.92E-20 |
| 50 | 119564 | F | 50 | 141993 | 0 | 1.92E-20 |
| 50 | 141993 | F | 50 | 150880 | 0 | 1.92E-20 |
| 50 | 188358 | F | 50 | 188368 | 0 | 1.92E-20 |
| 49 | 162835 | F | 49 | 243245 | 0 | 7.67E-20 |
| 52 | 69445 | P | 52 | 281573 | -1 | 1.87E-19 |
| 52 | 88665 | P | 52 | 206531 | -1 | 1.87E-19 |
| 52 | 88665 | P | 52 | 269197 | -1 | 1.87E-19 |
| 52 | 109048 | P | 52 | 281573 | -1 | 1.87E-19 |
| 52 | 140364 | P | 52 | 281573 | -1 | 1.87E-19 |
| 52 | 206531 | P | 52 | 292547 | -1 | 1.87E-19 |
| 52 | 269197 | P | 52 | 292547 | -1 | 1.87E-19 |
| 48 | 27999 | F | 48 | 265988 | 0 | 3.07E-19 |
| 57 | 288656 | F | 57 | 288683 | -3 | 9.24E-19 |
| 56 | 94619 | P | 56 | 282536 | -3 | 3.50E-18 |
| 55 | 67962 | F | 55 | 259994 | -3 | 1.33E-17 |
| 55 | 107565 | F | 55 | 259994 | -3 | 1.33E-17 |
| 55 | 138881 | F | 55 | 259994 | -3 | 1.33E-17 |
| 45 | 198706 | P | 45 | 281312 | 0 | 1.96E-17 |
| 54 | 198760 | P | 54 | 232996 | -3 | 5.01E-17 |
| 53 | 283768 | F | 53 | 283864 | -3 | 1.89E-16 |
| 43 | 88723 | F | 43 | 292235 | 0 | 3.14E-16 |
| 43 | 292235 | F | 43 | 292605 | 0 | 3.14E-16 |
| 52 | 72198 | P | 52 | 167604 | -3 | 7.15E-16 |
| 52 | 111801 | P | 52 | 167604 | -3 | 7.15E-16 |
| 52 | 143117 | P | 52 | 167604 | -3 | 7.15E-16 |
| 49 | 58941 | F | 49 | 232375 | -2 | 8.11E-16 |
| 49 | 129860 | F | 49 | 232375 | -2 | 8.11E-16 |
| 49 | 161176 | F | 49 | 232375 | -2 | 8.11E-16 |
| 45 | 198207 | P | 45 | 279269 | -1 | 2.65E-15 |
| 50 | 288669 | F | 50 | 288696 | -3 | 1.01E-14 |
| 40 | 69457 | P | 40 | 281573 | 0 | 2.01E-14 |
| 40 | 98949 | P | 40 | 107403 | 0 | 2.01E-14 |
| 40 | 107297 | F | 40 | 251675 | 0 | 2.01E-14 |
| 40 | 109060 | P | 40 | 281573 | 0 | 2.01E-14 |
| 40 | 140376 | P | 40 | 281573 | 0 | 2.01E-14 |
| 40 | 188358 | F | 40 | 188378 | 0 | 2.01E-14 |
| 49 | 102314 | F | 49 | 114721 | -3 | 3.81E-14 |
| 49 | 102314 | F | 49 | 146037 | -3 | 3.81E-14 |
| 43 | 47292 | F | 43 | 265937 | -1 | 4.05E-14 |
| 43 | 69555 | F | 43 | 88737 | -1 | 4.05E-14 |
| 43 | 69555 | F | 43 | 292619 | -1 | 4.05E-14 |
| 43 | 88737 | F | 43 | 109158 | -1 | 4.05E-14 |
| 43 | 88737 | F | 43 | 140474 | -1 | 4.05E-14 |
| 43 | 109158 | F | 43 | 292619 | -1 | 4.05E-14 |
| 43 | 140474 | F | 43 | 292619 | -1 | 4.05E-14 |
| 48 | 259821 | F | 48 | 263328 | -3 | 1.43E-13 |
| 42 | 48680 | P | 42 | 266447 | -1 | 1.58E-13 |
| 42 | 193717 | F | 42 | 225770 | -1 | 1.58E-13 |
| 45 | 94630 | P | 45 | 282536 | -2 | 1.75E-13 |
| 45 | 102530 | F | 45 | 114939 | -2 | 1.75E-13 |
| 45 | 102530 | F | 45 | 146255 | -2 | 1.75E-13 |
| 45 | 283666 | P | 45 | 292236 | -2 | 1.75E-13 |
| 47 | 71227 | F | 47 | 266090 | -3 | 5.37E-13 |
| 47 | 110830 | F | 47 | 266090 | -3 | 5.37E-13 |
| 47 | 142146 | F | 47 | 266090 | -3 | 5.37E-13 |
| 41 | 47260 | P | 41 | 178281 | -1 | 6.18E-13 |
| 41 | 283780 | F | 41 | 283876 | -1 | 6.18E-13 |
| 37 | 9912 | F | 37 | 235339 | 0 | 1.29E-12 |
| 37 | 79383 | F | 37 | 101111 | 0 | 1.29E-12 |
| 46 | 118553 | P | 46 | 245193 | -3 | 2.01E-12 |
| 46 | 149869 | P | 46 | 245193 | -3 | 2.01E-12 |
| 40 | 71252 | F | 40 | 178286 | -1 | 2.41E-12 |
| 40 | 110855 | F | 40 | 178286 | -1 | 2.41E-12 |
| 40 | 142171 | F | 40 | 178286 | -1 | 2.41E-12 |
| 43 | 192357 | F | 43 | 240506 | -2 | 2.55E-12 |
| 36 | 47260 | P | 36 | 71252 | 0 | 5.14E-12 |
| 36 | 47260 | P | 36 | 110855 | 0 | 5.14E-12 |
| 36 | 47260 | P | 36 | 142171 | 0 | 5.14E-12 |
| 36 | 69566 | P | 36 | 283622 | 0 | 5.14E-12 |
| 36 | 88703 | P | 36 | 283652 | 0 | 5.14E-12 |
| 36 | 109169 | P | 36 | 283622 | 0 | 5.14E-12 |
| 36 | 140485 | P | 36 | 283622 | 0 | 5.14E-12 |
| 36 | 283652 | P | 36 | 292585 | 0 | 5.14E-12 |
| 45 | 101666 | F | 45 | 114057 | -3 | 7.52E-12 |
| 45 | 101666 | F | 45 | 145373 | -3 | 7.52E-12 |
| 39 | 288680 | F | 39 | 288707 | -1 | 9.40E-12 |
| 42 | 88724 | P | 42 | 283669 | -2 | 9.73E-12 |
| 42 | 283669 | P | 42 | 292606 | -2 | 9.73E-12 |
| 35 | 29236 | P | 35 | 236911 | 0 | 2.06E-11 |
| 35 | 76142 | P | 35 | 281631 | 0 | 2.06E-11 |
| 35 | 125996 | F | 35 | 208106 | 0 | 2.06E-11 |
| 35 | 125996 | F | 35 | 270772 | 0 | 2.06E-11 |
| 35 | 157312 | F | 35 | 208106 | 0 | 2.06E-11 |
| 35 | 157312 | F | 35 | 270772 | 0 | 2.06E-11 |
| 38 | 26893 | F | 38 | 58890 | -1 | 3.67E-11 |
| 38 | 26893 | F | 38 | 129809 | -1 | 3.67E-11 |
| 38 | 26893 | F | 38 | 161125 | -1 | 3.67E-11 |
| 38 | 26948 | F | 38 | 58941 | -1 | 3.67E-11 |
| 38 | 26948 | F | 38 | 129860 | -1 | 3.67E-11 |
| 38 | 26948 | F | 38 | 161176 | -1 | 3.67E-11 |
| 41 | 100974 | F | 41 | 198613 | -2 | 3.71E-11 |
| 34 | 88511 | P | 34 | 206478 | 0 | 8.23E-11 |
| 34 | 88511 | P | 34 | 269144 | 0 | 8.23E-11 |
| 34 | 121349 | F | 34 | 212472 | 0 | 8.23E-11 |
| 34 | 152665 | F | 34 | 212472 | 0 | 8.23E-11 |
| 34 | 206478 | P | 34 | 292393 | 0 | 8.23E-11 |
| 34 | 269144 | P | 34 | 292393 | 0 | 8.23E-11 |
| 43 | 259842 | F | 43 | 263349 | -3 | 1.05E-10 |
| 43 | 283767 | F | 43 | 283838 | -3 | 1.05E-10 |
| 37 | 239973 | F | 37 | 240044 | -1 | 1.43E-10 |
| 33 | 67980 | F | 33 | 260012 | 0 | 3.29E-10 |
| 33 | 107583 | F | 33 | 260012 | 0 | 3.29E-10 |
| 33 | 138899 | F | 33 | 260012 | 0 | 3.29E-10 |
| 33 | 183997 | F | 33 | 204964 | 0 | 3.29E-10 |
| 33 | 183997 | F | 33 | 267630 | 0 | 3.29E-10 |
| 33 | 283788 | F | 33 | 283884 | 0 | 3.29E-10 |
| 42 | 12714 | F | 42 | 47436 | -3 | 3.89E-10 |
| 39 | 62320 | P | 39 | 214064 | -2 | 5.36E-10 |
| 39 | 133239 | P | 39 | 214064 | -2 | 5.36E-10 |
| 36 | 54818 | P | 36 | 180926 | -1 | 5.56E-10 |
| 36 | 72191 | P | 36 | 100739 | -1 | 5.56E-10 |
| 36 | 100739 | P | 36 | 111794 | -1 | 5.56E-10 |
| 36 | 100739 | P | 36 | 143110 | -1 | 5.56E-10 |
| 38 | 58890 | F | 38 | 232320 | -2 | 2.03E-09 |
| 38 | 88666 | F | 38 | 166182 | -2 | 2.03E-09 |
| 38 | 129809 | F | 38 | 232320 | -2 | 2.03E-09 |
| 38 | 161125 | F | 38 | 232320 | -2 | 2.03E-09 |
| 38 | 166182 | F | 38 | 292548 | -2 | 2.03E-09 |
| 35 | 101251 | P | 35 | 214065 | -1 | 2.16E-09 |
| 31 | 398 | F | 31 | 170018 | 0 | 5.27E-09 |
| 31 | 27863 | P | 31 | 258259 | 0 | 5.27E-09 |
| 37 | 275084 | F | 37 | 275261 | -2 | 7.71E-09 |
| 34 | 92731 | F | 34 | 92785 | -1 | 8.40E-09 |
| 34 | 113555 | F | 34 | 113587 | -1 | 8.40E-09 |
| 34 | 113555 | F | 34 | 144903 | -1 | 8.40E-09 |
| 34 | 113587 | F | 34 | 144871 | -1 | 8.40E-09 |
| 34 | 144871 | F | 34 | 144903 | -1 | 8.40E-09 |
| 30 | 178202 | P | 30 | 228325 | 0 | 2.11E-08 |
| 30 | 188358 | F | 30 | 188388 | 0 | 2.11E-08 |
| 30 | 228325 | P | 30 | 258375 | 0 | 2.11E-08 |
| 33 | 286390 | F | 33 | 286405 | -1 | 3.26E-08 |
| 38 | 88680 | F | 38 | 166196 | -3 | 7.32E-08 |
| 38 | 166182 | P | 38 | 206544 | -3 | 7.32E-08 |
| 38 | 166182 | P | 38 | 269210 | -3 | 7.32E-08 |
| 38 | 166196 | F | 38 | 292562 | -3 | 7.32E-08 |
| 38 | 228348 | P | 38 | 244809 | -3 | 7.32E-08 |
| 35 | 72215 | P | 35 | 167604 | -2 | 1.10E-07 |
| 35 | 111818 | P | 35 | 167604 | -2 | 1.10E-07 |
| 35 | 143134 | P | 35 | 167604 | -2 | 1.10E-07 |
| 32 | 69151 | F | 32 | 201670 | -1 | 1.26E-07 |
| 32 | 71837 | P | 32 | 119163 | -1 | 1.26E-07 |
| 32 | 71837 | P | 32 | 150479 | -1 | 1.26E-07 |
| 32 | 88748 | P | 32 | 283626 | -1 | 1.26E-07 |
| 32 | 108754 | F | 32 | 201670 | -1 | 1.26E-07 |
| 32 | 111440 | P | 32 | 119163 | -1 | 1.26E-07 |
| 32 | 111440 | P | 32 | 150479 | -1 | 1.26E-07 |
| 32 | 119163 | P | 32 | 142756 | -1 | 1.26E-07 |
| 32 | 140070 | F | 32 | 201670 | -1 | 1.26E-07 |
| 32 | 142756 | P | 32 | 150479 | -1 | 1.26E-07 |
| 32 | 206455 | P | 32 | 292220 | -1 | 1.26E-07 |
| 32 | 269121 | P | 32 | 292220 | -1 | 1.26E-07 |
| 32 | 283626 | P | 32 | 292630 | -1 | 1.26E-07 |
| 32 | 283679 | P | 32 | 292236 | -1 | 1.26E-07 |
| 32 | 283679 | P | 32 | 292606 | -1 | 1.26E-07 |
| 37 | 8996 | F | 37 | 20896 | -3 | 2.70E-07 |
| 37 | 82644 | F | 37 | 266446 | -3 | 2.70E-07 |
| 37 | 166196 | P | 37 | 206531 | -3 | 2.70E-07 |
| 37 | 166196 | P | 37 | 269197 | -3 | 2.70E-07 |
| 37 | 249761 | P | 37 | 266113 | -3 | 2.70E-07 |
| 34 | 22372 | F | 34 | 22429 | -2 | 4.16E-07 |
| 34 | 178281 | P | 34 | 265912 | -2 | 4.16E-07 |
| 34 | 283839 | F | 34 | 283864 | -2 | 4.16E-07 |
| 31 | 69621 | F | 31 | 88692 | -1 | 4.90E-07 |
| 31 | 69621 | F | 31 | 292574 | -1 | 4.90E-07 |
| 31 | 71270 | F | 31 | 243942 | -1 | 4.90E-07 |
| 31 | 88692 | F | 31 | 109224 | -1 | 4.90E-07 |
| 31 | 88692 | F | 31 | 140540 | -1 | 4.90E-07 |
| 31 | 99552 | F | 31 | 99561 | -1 | 4.90E-07 |
| 31 | 104641 | P | 31 | 274884 | -1 | 4.90E-07 |
| 31 | 107519 | F | 31 | 259951 | -1 | 4.90E-07 |
| 31 | 109224 | F | 31 | 292574 | -1 | 4.90E-07 |
| 31 | 110873 | F | 31 | 243942 | -1 | 4.90E-07 |
| 31 | 140540 | F | 31 | 292574 | -1 | 4.90E-07 |
| 31 | 142189 | F | 31 | 243942 | -1 | 4.90E-07 |
| 31 | 182874 | F | 31 | 262040 | -1 | 4.90E-07 |
| 31 | 275078 | F | 31 | 275114 | -1 | 4.90E-07 |
| 31 | 275142 | F | 31 | 275316 | -1 | 4.90E-07 |
| 36 | 71517 | P | 36 | 119434 | -3 | 9.92E-07 |
| 36 | 71517 | P | 36 | 150750 | -3 | 9.92E-07 |
| 36 | 88851 | F | 36 | 278850 | -3 | 9.92E-07 |
| 36 | 111120 | P | 36 | 119434 | -3 | 9.92E-07 |
| 36 | 111120 | P | 36 | 150750 | -3 | 9.92E-07 |
| 36 | 119434 | P | 36 | 142436 | -3 | 9.92E-07 |
| 36 | 142436 | P | 36 | 150750 | -3 | 9.92E-07 |
| 36 | 278850 | F | 36 | 292733 | -3 | 9.92E-07 |
| 30 | 3838 | P | 30 | 91675 | -1 | 1.90E-06 |
| 30 | 28449 | P | 30 | 207558 | -1 | 1.90E-06 |
| 30 | 28449 | P | 30 | 270224 | -1 | 1.90E-06 |
| 30 | 31207 | P | 30 | 77829 | -1 | 1.90E-06 |
| 30 | 69554 | P | 30 | 283669 | -1 | 1.90E-06 |
| 30 | 71690 | P | 30 | 119265 | -1 | 1.90E-06 |
| 30 | 71690 | P | 30 | 150581 | -1 | 1.90E-06 |
| 30 | 72199 | P | 30 | 100737 | -1 | 1.90E-06 |
| 30 | 89224 | F | 30 | 89231 | -1 | 1.90E-06 |
| 30 | 89224 | F | 30 | 293113 | -1 | 1.90E-06 |
| 30 | 89231 | F | 30 | 293106 | -1 | 1.90E-06 |
| 30 | 103835 | F | 30 | 116190 | -1 | 1.90E-06 |
| 30 | 103835 | F | 30 | 147506 | -1 | 1.90E-06 |
| 30 | 109157 | P | 30 | 283669 | -1 | 1.90E-06 |
| 30 | 111293 | P | 30 | 119265 | -1 | 1.90E-06 |
| 30 | 111293 | P | 30 | 150581 | -1 | 1.90E-06 |
| 30 | 119265 | P | 30 | 142609 | -1 | 1.90E-06 |
| 30 | 125870 | P | 30 | 283638 | -1 | 1.90E-06 |
| 30 | 140473 | P | 30 | 283669 | -1 | 1.90E-06 |
| 30 | 142609 | P | 30 | 150581 | -1 | 1.90E-06 |
| 30 | 157186 | P | 30 | 283638 | -1 | 1.90E-06 |
| 30 | 243911 | F | 30 | 258437 | -1 | 1.90E-06 |
| 30 | 293106 | F | 30 | 293113 | -1 | 1.90E-06 |
| 35 | 62323 | F | 35 | 101251 | -3 | 3.64E-06 |
| 35 | 69156 | F | 35 | 274826 | -3 | 3.64E-06 |
| 35 | 101251 | F | 35 | 133242 | -3 | 3.64E-06 |
| 35 | 101879 | F | 35 | 114270 | -3 | 3.64E-06 |
| 35 | 101879 | F | 35 | 145586 | -3 | 3.64E-06 |
| 35 | 107419 | P | 35 | 120638 | -3 | 3.64E-06 |
| 35 | 107419 | P | 35 | 151954 | -3 | 3.64E-06 |
| 35 | 108759 | F | 35 | 274826 | -3 | 3.64E-06 |
| 35 | 140075 | F | 35 | 274826 | -3 | 3.64E-06 |
| 32 | 67859 | F | 32 | 67894 | -2 | 5.88E-06 |
| 32 | 67859 | F | 32 | 138813 | -2 | 5.88E-06 |
| 32 | 67894 | F | 32 | 138778 | -2 | 5.88E-06 |
| 32 | 126168 | F | 32 | 282651 | -2 | 5.88E-06 |
| 32 | 138778 | F | 32 | 138813 | -2 | 5.88E-06 |
| 32 | 157484 | F | 32 | 282651 | -2 | 5.88E-06 |
| 32 | 195363 | F | 32 | 195433 | -2 | 5.88E-06 |
| 32 | 247720 | P | 32 | 278299 | -2 | 5.88E-06 |
| 34 | 69217 | F | 34 | 250472 | -3 | 1.33E-05 |
| 34 | 108820 | F | 34 | 250472 | -3 | 1.33E-05 |
| 34 | 140136 | F | 34 | 250472 | -3 | 1.33E-05 |
| 34 | 249768 | F | 34 | 258252 | -3 | 1.33E-05 |
| 31 | 454 | P | 31 | 169997 | -2 | 2.20E-05 |
| 31 | 35147 | F | 31 | 249775 | -2 | 2.20E-05 |
| 31 | 165315 | P | 31 | 280198 | -2 | 2.20E-05 |
| 31 | 182983 | P | 31 | 281233 | -2 | 2.20E-05 |
| 31 | 292177 | F | 31 | 292215 | -2 | 2.20E-05 |
| 33 | 27863 | P | 33 | 35145 | -3 | 4.85E-05 |
| 33 | 71483 | P | 33 | 119471 | -3 | 4.85E-05 |
| 33 | 71483 | P | 33 | 150787 | -3 | 4.85E-05 |
| 33 | 102342 | F | 33 | 114749 | -3 | 4.85E-05 |
| 33 | 102342 | F | 33 | 146065 | -3 | 4.85E-05 |
| 33 | 111086 | P | 33 | 119471 | -3 | 4.85E-05 |
| 33 | 111086 | P | 33 | 150787 | -3 | 4.85E-05 |
| 33 | 119471 | P | 33 | 142402 | -3 | 4.85E-05 |
| 33 | 142402 | P | 33 | 150787 | -3 | 4.85E-05 |
| 30 | 2612 | F | 30 | 223176 | -2 | 8.25E-05 |
| 30 | 3331 | F | 30 | 43020 | -2 | 8.25E-05 |
| 30 | 17911 | F | 30 | 61441 | -2 | 8.25E-05 |
| 30 | 17911 | F | 30 | 132360 | -2 | 8.25E-05 |
| 30 | 22774 | F | 30 | 281196 | -2 | 8.25E-05 |
| 30 | 27958 | F | 30 | 47304 | -2 | 8.25E-05 |
| 30 | 35152 | F | 30 | 258264 | -2 | 8.25E-05 |
| 30 | 42330 | P | 30 | 43528 | -2 | 8.25E-05 |
| 30 | 47309 | F | 30 | 265954 | -2 | 8.25E-05 |
| 30 | 66840 | F | 30 | 82853 | -2 | 8.25E-05 |
| 30 | 69555 | F | 30 | 292249 | -2 | 8.25E-05 |
| 30 | 70005 | F | 30 | 202065 | -2 | 8.25E-05 |
| 30 | 71252 | P | 30 | 265911 | -2 | 8.25E-05 |
| 30 | 72224 | F | 30 | 281052 | -2 | 8.25E-05 |
| 30 | 72588 | P | 30 | 88570 | -2 | 8.25E-05 |
| 30 | 72588 | P | 30 | 292452 | -2 | 8.25E-05 |
| 30 | 82853 | F | 30 | 137759 | -2 | 8.25E-05 |
| 30 | 88571 | P | 30 | 112190 | -2 | 8.25E-05 |
| 30 | 88571 | P | 30 | 143506 | -2 | 8.25E-05 |
| 30 | 100737 | F | 30 | 167625 | -2 | 8.25E-05 |
| 30 | 109158 | F | 30 | 292249 | -2 | 8.25E-05 |
| 30 | 109608 | F | 30 | 202065 | -2 | 8.25E-05 |
| 30 | 110855 | P | 30 | 265911 | -2 | 8.25E-05 |
| 30 | 111827 | F | 30 | 281052 | -2 | 8.25E-05 |
| 30 | 112191 | P | 30 | 292452 | -2 | 8.25E-05 |
| 30 | 140474 | F | 30 | 292249 | -2 | 8.25E-05 |
| 30 | 140924 | F | 30 | 202065 | -2 | 8.25E-05 |
| 30 | 142171 | P | 30 | 265911 | -2 | 8.25E-05 |
| 30 | 143143 | F | 30 | 281052 | -2 | 8.25E-05 |
| 30 | 143507 | P | 30 | 292452 | -2 | 8.25E-05 |
| 30 | 167600 | P | 30 | 281052 | -2 | 8.25E-05 |
| 30 | 178229 | F | 30 | 243900 | -2 | 8.25E-05 |
| 30 | 193934 | P | 30 | 204935 | -2 | 8.25E-05 |
| 30 | 193934 | P | 30 | 267601 | -2 | 8.25E-05 |
| 30 | 237448 | P | 30 | 239819 | -2 | 8.25E-05 |
| 30 | 249780 | F | 30 | 258264 | -2 | 8.25E-05 |
| 30 | 283851 | F | 30 | 283876 | -2 | 8.25E-05 |
| 32 | 20903 | P | 32 | 26432 | -3 | 1.76E-04 |
| 32 | 23028 | P | 32 | 246950 | -3 | 1.76E-04 |
| 32 | 67862 | F | 32 | 67885 | -3 | 1.76E-04 |
| 32 | 67862 | F | 32 | 138804 | -3 | 1.76E-04 |
| 32 | 67885 | F | 32 | 138781 | -3 | 1.76E-04 |
| 32 | 67947 | F | 32 | 259979 | -3 | 1.76E-04 |
| 32 | 104077 | F | 32 | 258320 | -3 | 1.76E-04 |
| 32 | 107550 | F | 32 | 259979 | -3 | 1.76E-04 |
| 32 | 125936 | P | 32 | 274797 | -3 | 1.76E-04 |

**Table S10 DNA transfer of *S. divaricata* organelle genomes.**

| Group | Length | Chloroplast genome | | mitogenome | | E-value | Chloroplast coding region | Chloroplast coding length | Chloroplast coding genes | Mitogenome | Mitogenome coding length | Mitogenome coding genes | Direction (mitogenome) |  |
| --- | --- | --- | --- | --- | --- | --- | --- | --- | --- | --- | --- | --- | --- | --- |
|  |  |  |  |  |  |  |  |  |  | coding region |  |  |  |  |
|  |  | Start | End | Start | End |  |  |  |  |  |  |  |  |  |
| Ⅰ | 6813 | 101995 | 108807 | 119569 | 112758 | 0 | *rrn*16S-*trn*N-GUU | 5477 | Partial (*rrn*16S) | *cox*2-*rrn*26 | 181 | Complete (*trn*R-ACG, *rrn*4.5) | Positive |  |
|  |  | 139040 | 132228 |  |  |  |  |  | Complete *(trn*I-GAU, *trn*A-UGC, rrn23S, rrn4.5S, rrn5S, trnR-ACG) |  |  |  |  |  |
|  |  |  |  |  |  |  |  |  |  |  |  |  |  |  |
|  |  | 101995 | 108807 | 150885 | 144074 |  |  |  |  | *cob*-*rrn*5 | 79 | Complete (*trn*R-ACG) | Negative |  |
|  |  |  |  |  |  |  |  |  |  |  |  |  |  |  |
|  |  |  |  |  |  |  |  |  |  |  |  |  |  |  |
|  |  | 139040 | 132228 |  |  |  |  |  |  |  |  |  |  |  |
|  |  |  |  |  |  |  |  |  |  |  |  |  |  |  |
| Ⅱ | 888 | 139020 | 139883 | 71079 | 70221 | 1.75E-85 | *rrn*16S | 864 | Partial (*rrn*16S) | *nad*7-*rpl*10 | 0 | Null | Null |  |
|  |  | 102015 | 101152 |  |  |  |  |  |  |  |  |  |  |  |
|  |  | 139020 | 139883 | 110682 | 109824 |  |  |  |  | *trn*R-ACG-*rrn*26 | 0 | Null | Null |  |
|  |  | 102015 | 101152 |  |  |  |  |  |  |  |  |  |  |  |
|  |  | 139020 | 139883 | 141998 | 141140 |  |  |  |  | *rrn*18 | 859 | Partial (*rrn*18) | Negative |  |
|  |  | 102015 | 101152 |  |  |  |  |  |  |  |  |  |  |  |
| Ⅲ | 104 | 108704 | 108807 | 73257 | 73155 | 2.39E-44 | *trn*R-ACG-*trn*N-GUU | 0 | Null | *nad*7-*rpl*10 | 0 | Null | Null |  |
|  |  | 132331 | 132228 |  |  |  |  |  |  |  |  |  |  |  |
| Ⅳ | 82 | 109185 | 109266 | 34749 | 34668 | 2.43E-34 | *trn*R-ACG-*ycf*1 | 72 | Complete (*trn*N-GUU) | *trn*Y-GUA-*trn*C-GCA | 72 | Complete (*trn*N-GUU) | Negative |  |
|  |  | 131769 | 131850 |  |  |  |  |  |  |  |  |  |  |  |
| Ⅴ | 79 | 55 | 133 | 228770 | 228692 | 2.43E-34 | *trn*L-CAA-*psb*A | 75 | Complete (*trn*H-GUG) | *atp*6-*trn*E-UUC | 74 | Complete (*trn*H-GUG) | Negative |  |
| Ⅵ | 78 | 86835 | 86909 | 246004 | 246081 | 5.32E-21 | *rpl*23-*trn*I-CAU | 74 | Complete (*trn*I-CAU) | *rps*13-*trn*I-CAU | 77 | Complete (*trn*I-CAU) | Positive |  |
| Ⅶ | 524 | 38935 | 39456 | 263233 | 262718 | 0 | *psa*B | 522 | Partial (*psa*B) | *trn*M-CAU-*rps*3 | 0 | Null | Negative |  |
| Ⅷ | 379 | 66613 | 66985 | 28759 | 28389 | 7.96E-99 | *pet*L-*trn*P-UGG | 188 | Complete (*pet*G, trnW-CCA) | *trn*C-GCA-*nad*9 | 74 | Complete (*trn*W-CCA) | Negative |  |
|  |  |  |  |  |  |  |  |  |  |  |  |  |  |  |
| Ⅸ | 214 | 25512 | 25725 | 215088 | 214875 | 4.86E-86 | *rpo*B | 214 | Partial (*rpo*B) | *nad*6-*trn*G-GCC | 0 | Null | Null |  |
| Ⅹ | 171 | 30970 | 31135 | 54311 | 54481 | 1.84E-50 | *psb*M-*trn*Y-GUA | 75 | Complete (*trn*D-GUC) | *cox*3-*rpl*10 | 74 | Complete (*trn*D-GUC) | Positive |  |

**Table S11 The dN, dS of the common genes of *S. divaricata* mitogenome.**

| Gene | dN/dS | dN | dS |
| --- | --- | --- | --- |
| atp1 | 0.216006 | 0.0278 | 0.1287 |
| atp1 | 0.091848 | 0.032 | 0.3484 |
| atp1 | 0.076547 | 0.0282 | 0.3684 |
| atp1 | 0.217822 | 0.0264 | 0.1212 |
| atp1 | 0.228571 | 0.0032 | 0.014 |
| atp1 | 0.073184 | 0.0268 | 0.3662 |
| atp1 | 0.217822 | 0.0264 | 0.1212 |
| atp1 | 0.228571 | 0.0032 | 0.014 |
| atp1 | 0.073184 | 0.0268 | 0.3662 |
| atp1 | 0.217822 | 0.0264 | 0.1212 |
| atp1 | 0.228571 | 0.0032 | 0.014 |
| atp1 | 0.073184 | 0.0268 | 0.3662 |
| atp4 | 0.895197 | 0.082 | 0.0916 |
| atp4 | 0.895355 | 0.1677 | 0.1873 |
| atp4 | 0.820134 | 0.1222 | 0.149 |
| atp4 | 0.86966 | 0.0794 | 0.0913 |
| atp4 | 0.844909 | 0.1253 | 0.1483 |
| atp4 | 0.902198 | 0.0821 | 0.091 |
| atp4 | 0.828938 | 0.1226 | 0.1479 |
| atp4 | 0.902198 | 0.0821 | 0.091 |
| atp4 | 0.828938 | 0.1226 | 0.1479 |
| atp6 | 0.379271 | 0.0333 | 0.0878 |
| atp6 | 0.425435 | 0.0562 | 0.1321 |
| atp6 | 0.37619 | 0.0474 | 0.126 |
| atp6 | 0.32666 | 0.031 | 0.0949 |
| atp6 | 0.06006 | 0.002 | 0.0333 |
| atp6 | 0.334825 | 0.0449 | 0.1341 |
| atp6 | 0.270624 | 0.0269 | 0.0994 |
| atp6 | 0.16095 | 0.0061 | 0.0379 |
| atp6 | 0.338843 | 0.0451 | 0.1331 |
| atp6 | 0.166667 | 0.004 | 0.024 |
| atp6 | 0.270624 | 0.0269 | 0.0994 |
| atp6 | 0.16095 | 0.0061 | 0.0379 |
| atp6 | 0.338843 | 0.0451 | 0.1331 |
| atp6 | 0.166667 | 0.004 | 0.024 |
| atp9 | 0.158579 | 0.083 | 0.5234 |
| atp9 | 0.02955 | 0.0434 | 1.4687 |
| atp9 | 0.120461 | 0.0919 | 0.7629 |
| atp9 | 0.133864 | 0.074 | 0.5528 |
| atp9 | 0.105715 | 0.0849 | 0.8031 |
| atp9 | 0.133864 | 0.074 | 0.5528 |
| atp9 | 0.105715 | 0.0849 | 0.8031 |
| atp9 | 0.141515 | 0.0738 | 0.5215 |
| atp9 | 0.111653 | 0.0847 | 0.7586 |
| ccmB | 2.133621 | 0.0495 | 0.0232 |
| ccmB | 1.213549 | 0.1648 | 0.1358 |
| ccmB | 1.272308 | 0.1654 | 0.13 |
| ccmB | 2.133621 | 0.0495 | 0.0232 |
| ccmB | 1.272308 | 0.1654 | 0.13 |
| ccmB | 1.710345 | 0.0496 | 0.029 |
| ccmB | 1.239762 | 0.1665 | 0.1343 |
| ccmB | 1.410256 | 0.0495 | 0.0351 |
| ccmB | 1.170423 | 0.1662 | 0.142 |
| ccmC | 0.658182 | 0.0543 | 0.0825 |
| ccmC | 0.709456 | 0.1343 | 0.1893 |
| ccmC | 0.967765 | 0.1381 | 0.1427 |
| ccmC | 0.633028 | 0.0483 | 0.0763 |
| ccmC | 1.14 | 0.0057 | 0.005 |
| ccmC | 0.935767 | 0.1384 | 0.1479 |
| ccmC | 0.591912 | 0.0483 | 0.0816 |
| ccmC | 0.564356 | 0.0057 | 0.0101 |
| ccmC | 0.902932 | 0.1386 | 0.1535 |
| ccmC | 0.591912 | 0.0483 | 0.0816 |
| ccmC | 0.564356 | 0.0057 | 0.0101 |
| ccmC | 0.902932 | 0.1386 | 0.1535 |
| cob | 0.529524 | 0.0278 | 0.0525 |
| cob | 0.226693 | 0.0462 | 0.2038 |
| cob | 0.257525 | 0.0462 | 0.1794 |
| cob | 0.429078 | 0.0242 | 0.0564 |
| cob | 0.944444 | 0.0034 | 0.0036 |
| cob | 0.263773 | 0.0474 | 0.1797 |
| cob | 0.333333 | 0.0266 | 0.0798 |
| cob | 0.278788 | 0.0092 | 0.033 |
| cob | 0.246313 | 0.0451 | 0.1831 |
| cob | 0.235495 | 0.0069 | 0.0293 |
| cob | 0.363409 | 0.029 | 0.0798 |
| cob | 0.278788 | 0.0092 | 0.033 |
| cob | 0.246179 | 0.0451 | 0.1832 |
| cob | 0.235495 | 0.0069 | 0.0293 |
| matR | 0.493261 | 0.0366 | 0.0742 |
| matR | 0.502994 | 0.0924 | 0.1837 |
| matR | 0.547771 | 0.0774 | 0.1413 |
| matR | 0.582569 | 0.0381 | 0.0654 |
| matR | 0.358025 | 0.0029 | 0.0081 |
| matR | 0.566203 | 0.0774 | 0.1367 |
| matR | 0.529167 | 0.0381 | 0.072 |
| matR | 0.47541 | 0.0058 | 0.0122 |
| matR | 0.566738 | 0.0794 | 0.1401 |
| matR | 0.716049 | 0.0058 | 0.0081 |
| matR | 0.529167 | 0.0381 | 0.072 |
| matR | 0.540984 | 0.0066 | 0.0122 |
| matR | 0.567857 | 0.0795 | 0.14 |
| matR | 0.814815 | 0.0066 | 0.0081 |
| nad3 | 0.181604 | 0.0154 | 0.0848 |
| nad3 | 0.357196 | 0.0479 | 0.1341 |
| nad3 | 0.677054 | 0.0478 | 0.0706 |
| nad3 | 0.089412 | 0.0076 | 0.085 |
| nad3 | 0.792373 | 0.0561 | 0.0708 |
| nad3 | 0.135453 | 0.0115 | 0.0849 |
| nad3 | 0.734088 | 0.0519 | 0.0707 |
| nad3 | 0.135453 | 0.0115 | 0.0849 |
| nad3 | 0.734088 | 0.0519 | 0.0707 |
| nad4L | 0.455927 | 0.015 | 0.0329 |
| nad4L | 0.250493 | 0.0254 | 0.1014 |
| nad4L | 0.095785 | 0.01 | 0.1044 |
| nad4L | 0.455927 | 0.015 | 0.0329 |
| nad4L | 0.095785 | 0.01 | 0.1044 |
| nad4L | 0.299401 | 0.015 | 0.0501 |
| nad4L | 0.080972 | 0.01 | 0.1235 |
| nad4L | 0.299401 | 0.015 | 0.0501 |
| nad4L | 0.080972 | 0.01 | 0.1235 |
| nad6 | 0.933735 | 0.0465 | 0.0498 |
| nad6 | 0.885071 | 0.0747 | 0.0844 |
| nad6 | 0.67107 | 0.0508 | 0.0757 |
| nad6 | 0.827402 | 0.0465 | 0.0562 |
| nad6 | 0.614545 | 0.0507 | 0.0825 |
| nad6 | 0.782918 | 0.044 | 0.0562 |
| nad6 | 0.396552 | 0.0023 | 0.0058 |
| nad6 | 0.644417 | 0.0531 | 0.0824 |
| nad6 | 0.198276 | 0.0023 | 0.0116 |
| nad6 | 0.815725 | 0.0332 | 0.0407 |
| nad6 | 0.628713 | 0.0127 | 0.0202 |
| nad6 | 0.669891 | 0.0554 | 0.0827 |
| nad6 | 0.48289 | 0.0127 | 0.0263 |
| nad6 | 0.722222 | 0.0104 | 0.0144 |
| nad9 | 1.043011 | 0.0291 | 0.0279 |
| nad9 | 0.830065 | 0.0381 | 0.0459 |
| nad9 | 0.437071 | 0.0191 | 0.0437 |
| nad9 | 1.399038 | 0.0291 | 0.0208 |
| nad9 | 0.695652 | 0.0048 | 0.0069 |
| nad9 | 0.533333 | 0.0192 | 0.036 |
| nad9 | 1.405797 | 0.0291 | 0.0207 |
| nad9 | 0.695652 | 0.0048 | 0.0069 |
| nad9 | 0.534819 | 0.0192 | 0.0359 |
| nad9 | 1.405797 | 0.0291 | 0.0207 |
| nad9 | 0.695652 | 0.0048 | 0.0069 |
| nad9 | 0.534819 | 0.0192 | 0.0359 |
| rps4 | 0.582797 | 0.0996 | 0.1709 |
| rps4 | 1.058912 | 0.2103 | 0.1986 |
| rps4 | 1.029503 | 0.2652 | 0.2576 |
| rps4 | 0.582797 | 0.0996 | 0.1709 |
| rps4 | 1.029503 | 0.2652 | 0.2576 |
| rps4 | 0.605386 | 0.1034 | 0.1708 |
| rps4 | 1.047748 | 0.2699 | 0.2576 |
| rps4 | 0.605386 | 0.1034 | 0.1708 |
| rps4 | 1.047748 | 0.2699 | 0.2576 |
| rps12 | 0.148828 | 0.0146 | 0.0981 |
| rps12 | 0.169837 | 0.05 | 0.2944 |
| rps12 | 0.247276 | 0.0522 | 0.2111 |
| rps12 | 0.16957 | 0.0146 | 0.0861 |
| rps12 | 0.264901 | 0.052 | 0.1963 |
| rps12 | 0.16957 | 0.0146 | 0.0861 |
| rps12 | 0.264901 | 0.052 | 0.1963 |
| rps12 | 0.16957 | 0.0146 | 0.0861 |
| rps12 | 0.264901 | 0.052 | 0.1963 |

**Table S12 RNA editing sites of *S. divaricata* organelle genomes.**

| Organelle | Position | Reference | Stand | Coverage-q25 | MeanQ | BaseCount[A,C,G,T] | AllSubs | Frequency | Pvalue | Region |
| --- | --- | --- | --- | --- | --- | --- | --- | --- | --- | --- |
| chloroplast | 5505 | G |  | 21 | 37.00 | [21, 0, 0, 0] | GA | 1.00 | 4.09E-11 | *rps*16 |
| chloroplast | 71350 | C |  | 22 | 35.91 | [15, 7, 0, 0] | CA | 0.68 | 9.18E-06 | *clp*P |
| mitochondrion | 156772 | C |  | 159 | 36.55 | [0, 1, 0, 158] | CT | 0.99 | 1.06E-90 | *nad*4 |
| mitochondrion | 125456 | C |  | 158 | 36.39 | [0, 3, 0, 155] | CT | 0.98 | 1.75E-86 | *nad*4 |
| mitochondrion | 125333 | C |  | 165 | 36.27 | [0, 6, 159, 0] | CG | 0.96 | 5.11E-86 | *nad*5 |
| mitochondrion | 156649 | G |  | 150 | 36.44 | [0, 140, 10, 0] | GC | 0.93 | 3.21E-72 | *nad*5 |
| mitochondrion | 156556 | G |  | 121 | 36.4 | [118, 0, 3, 0] | GA | 0.98 | 9.95E-65 | IGS(*nad*5-*cox*3) |
| mitochondrion | 125240 | G |  | 116 | 36.9 | [113, 0, 3, 0] | GA | 0.97 | 8.43E-62 | IGS(*nad*5-*cox*3) |
| mitochondrion | 141839 | T |  | 184 | 36.28 | [82, 1, 0, 101] | TA | 0.45 | 8.04E-29 | *cox*3 |
| mitochondrion | 70920 | T |  | 182 | 36.34 | [76, 0, 0, 106] | TA | 0.42 | 2.96E-26 | *cox*3 |
| mitochondrion | 91620 | C |  | 45 | 36.2 | [0, 0, 0, 45] | CT | 1 | 4.43E-25 | *cox*3 |
| mitochondrion | 110523 | T |  | 174 | 36.52 | [62, 1, 0, 111] | TA | 0.36 | 1.04E-20 | *cox*3 |
| mitochondrion | 150887 | C |  | 35 | 37 | [0, 0, 35, 0] | CG | 1 | 3.21E-19 | *cox*3 |
| mitochondrion | 119571 | C |  | 31 | 37 | [0, 0, 31, 0] | CG | 1 | 6.88E-17 | *cox*3 |
| mitochondrion | 281198 | C |  | 28 | 37 | [0, 0, 0, 28] | CT | 1 | 3.79E-15 | IGS(*cox*3-*trn*D-GUC) |
| mitochondrion | 91506 | G |  | 28 | 37 | [28, 0, 0, 0] | GA | 1 | 3.79E-15 | *rpl*10 |
| mitochondrion | 91544 | C |  | 28 | 36.57 | [0, 0, 0, 28] | CT | 1 | 3.79E-15 | IGS(*rpl*10-*nad*7) |
| mitochondrion | 91392 | C |  | 26 | 36.54 | [0, 0, 0, 26] | CT | 1 | 5.44E-14 | *nad*8 |
| mitochondrion | 98888 | G |  | 31 | 36.61 | [28, 0, 3, 0] | GA | 0.9 | 3.41E-13 | *nad*7 |
| mitochondrion | 91818 | G |  | 24 | 35 | [24, 0, 0, 0] | GA | 1 | 7.75E-13 | *nad*7 |
| mitochondrion | 53406 | G |  | 43 | 35.88 | [31, 0, 12, 0] | GA | 0.72 | 1.67E-12 | *nad*7 |
| mitochondrion | 259258 | G |  | 24 | 35 | [23, 0, 1, 0] | GA | 0.96 | 1.79E-11 | *nad*7 |
| mitochondrion | 125397 | G |  | 203 | 36.59 | [0, 37, 166, 0] | GC | 0.18 | 2.55E-11 | *nad*7 |
| mitochondrion | 263548 | G |  | 21 | 37 | [21, 0, 0, 0] | GA | 1 | 4.09E-11 | *nad*7 |
| mitochondrion | 52948 | C |  | 21 | 36.43 | [0, 0, 0, 21] | CT | 1 | 4.09E-11 | *atp*1 |
| mitochondrion | 78408 | G |  | 21 | 36.43 | [21, 0, 0, 0] | GA | 1 | 4.09E-11 | *atp1* |
| mitochondrion | 91367 | C |  | 21 | 35.86 | [0, 0, 0, 21] | CT | 1 | 4.09E-11 | *atp*1 |
| mitochondrion | 156713 | G |  | 194 | 36.32 | [0, 35, 159, 0] | GC | 0.18 | 1.09E-10 | *atp*1 |
| mitochondrion | 49703 | G |  | 19 | 36.37 | [19, 0, 0, 0] | GA | 1 | 5.66E-10 | *atp*1 |
| mitochondrion | 50052 | G |  | 19 | 37 | [19, 0, 0, 0] | GA | 1 | 5.66E-10 | *atp*1 |
| mitochondrion | 119572 | T |  | 18 | 35 | [0, 18, 0, 0] | TC | 1 | 2.09E-09 | *atp*8 |
| mitochondrion | 222184 | G |  | 18 | 37 | [18, 0, 0, 0] | GA | 1 | 2.09E-09 | *atp*8 |
| mitochondrion | 80439 | C |  | 18 | 36.33 | [0, 0, 0, 18] | CT | 1 | 2.09E-09 | IGS(*rrn*26-*trn*R-ACG) |
| mitochondrion | 53038 | C |  | 22 | 37 | [0, 2, 0, 20] | CT | 0.91 | 2.54E-09 | IGS(*rrn*4.5-*cox*2) |
| mitochondrion | 123556 | C |  | 17 | 36.29 | [0, 0, 0, 17] | CT | 1 | 7.71E-09 | IGS(*rrn*4.5-*cox*2) |
| mitochondrion | 53092 | C |  | 17 | 37 | [0, 0, 0, 17] | CT | 1 | 7.71E-09 | IGS(*rrn*4.5-*cox*2) |
| mitochondrion | 80173 | C |  | 17 | 37 | [0, 0, 0, 17] | CT | 1 | 7.71E-09 | IGS(*rrn*4.5-*cox*2) |
| mitochondrion | 156896 | A |  | 21 | 37 | [2, 19, 0, 0] | AC | 0.9 | 8.62E-09 | IGS(*rrn*4.5-*cox*2) |
| mitochondrion | 156899 | A |  | 22 | 35.91 | [3, 0, 0, 19] | AT | 0.86 | 1.94E-08 | IGS(*rrn*4.5-*cox*2) |
| mitochondrion | 53290 | C |  | 16 | 37 | [0, 0, 0, 16] | CT | 1 | 2.83E-08 | IGS(*rrn*4.5-*cox*2) |
| mitochondrion | 81410 | G |  | 16 | 37 | [16, 0, 0, 0] | GA | 1 | 2.83E-08 | IGS(*rrn*4.5-*cox*2) |
| mitochondrion | 79204 | C |  | 18 | 36.33 | [0, 1, 0, 17] | CT | 0.94 | 3.58E-08 | IGS(*rrn*4.5-*cox*2) |
| mitochondrion | 125580 | A |  | 23 | 36.48 | [4, 19, 0, 0] | AC | 0.83 | 3.66E-08 | IGS(*rrn*4.5-*cox*2) |
| mitochondrion | 52638 | G |  | 21 | 36.43 | [18, 0, 3, 0] | GA | 0.86 | 6.30E-08 | IGS(*rrn*4.5-*cox*2) |
| mitochondrion | 123751 | C |  | 15 | 35.4 | [0, 0, 0, 15] | CT | 1 | 1.03E-07 | IGS(*rrn*4.5-*cox*2) |
| mitochondrion | 154647 | G |  | 15 | 35.4 | [15, 0, 0, 0] | GA | 1 | 1.03E-07 | IGS(*rrn*4.5-*cox*2) |
| mitochondrion | 154657 | G |  | 15 | 36.2 | [15, 0, 0, 0] | GA | 1 | 1.03E-07 | IGS(*rrn*4.5-*cox*2) |
| mitochondrion | 16863 | C |  | 15 | 37 | [0, 0, 0, 15] | CT | 1 | 1.03E-07 | IGS(*rrn*4.5-*cox*2) |
| mitochondrion | 184406 | G |  | 15 | 37 | [15, 0, 0, 0] | GA | 1 | 1.03E-07 | IGS(*rrn*4.5-*cox*2) |
| mitochondrion | 232441 | C |  | 15 | 36.2 | [0, 0, 0, 15] | CT | 1 | 1.03E-07 | IGS(*rrn*4.5-*cox*2) |
| mitochondrion | 125583 | A |  | 22 | 37 | [4, 0, 0, 18] | AT | 0.82 | 1.15E-07 | *rrn*18 |
| mitochondrion | 50772 | G |  | 14 | 37 | [14, 0, 0, 0] | GA | 1 | 3.74E-07 | IGS(*trn*R-ACG-*cob*) |
| mitochondrion | 53280 | C |  | 14 | 37 | [0, 0, 0, 14] | CT | 1 | 3.74E-07 | IGS(*trn*R-ACG-*cob*) |
| mitochondrion | 58318 | G |  | 14 | 37 | [14, 0, 0, 0] | GA | 1 | 3.74E-07 | IGS(*trn*R-ACG-*cob*) |
| mitochondrion | 80240 | G |  | 14 | 37 | [14, 0, 0, 0] | GA | 1 | 3.74E-07 | *cox*1 |
| mitochondrion | 81455 | G |  | 19 | 35.74 | [16, 0, 3, 0] | GA | 0.84 | 6.46E-07 | *cox*1 |
| mitochondrion | 150886 | A |  | 38 | 36.37 | [19, 19, 0, 0] | AC | 0.5 | 1.26E-06 | *cox*1 |
| mitochondrion | 123331 | G |  | 13 | 37 | [13, 0, 0, 0] | GA | 1 | 1.35E-06 | *cox*1 |
| mitochondrion | 123341 | G |  | 13 | 36.08 | [13, 0, 0, 0] | GA | 1 | 1.35E-06 | *cox*1 |
| mitochondrion | 123644 | C |  | 13 | 36.08 | [0, 0, 0, 13] | CT | 1 | 1.35E-06 | *cox*1 |
| mitochondrion | 154872 | C |  | 13 | 36.08 | [0, 0, 0, 13] | CT | 1 | 1.35E-06 | *rps*7 |
| mitochondrion | 155067 | C |  | 13 | 37 | [0, 0, 0, 13] | CT | 1 | 1.35E-06 | *rps*7 |
| mitochondrion | 232894 | C |  | 13 | 36.08 | [0, 0, 0, 13] | CT | 1 | 1.35E-06 | *rps*7 |
| mitochondrion | 50584 | C |  | 13 | 37 | [0, 0, 0, 13] | CT | 1 | 1.35E-06 | *rps*7 |
| mitochondrion | 125450 | C |  | 167 | 36.57 | [0, 145, 0, 22] | CT | 0.13 | 1.45E-06 | *rps*7 |
| mitochondrion | 124815 | G |  | 12 | 37 | [12, 0, 0, 0] | GA | 1 | 4.81E-06 | IGS(*rps*7-*ccm*FN) |
| mitochondrion | 12984 | C |  | 12 | 37 | [0, 0, 0, 12] | CT | 1 | 4.81E-06 | IGS(*rps*7-*ccm*FN) |
| mitochondrion | 222201 | G |  | 12 | 35 | [12, 0, 0, 0] | GA | 1 | 4.81E-06 | *rpl*5 |
| mitochondrion | 156766 | G |  | 171 | 36.72 | [20, 0, 151, 0] | GA | 0.12 | 6.15E-06 | *nad*6 |
| mitochondrion | 123425 | G |  | 11 | 34.82 | [11, 0, 0, 0] | GA | 1 | 1.70E-05 | *nad*6 |
| mitochondrion | 124478 | C |  | 11 | 37 | [0, 0, 0, 11] | CT | 1 | 1.70E-05 | IGS(*nad*6-*trn*E-UUC) |
| mitochondrion | 154960 | G |  | 11 | 35.91 | [11, 0, 0, 0] | GA | 1 | 1.70E-05 | IGS(*trn*H-GUG-*atp*6) |
| mitochondrion | 222708 | G |  | 16 | 37 | [13, 0, 3, 0] | GA | 0.81 | 1.93E-05 | *atp*6 |
| mitochondrion | 150888 | T |  | 10 | 35.8 | [0, 10, 0, 0] | TC | 1 | 5.95E-05 | IGS(*trn*M-CAU-*rps*3) |
| mitochondrion | 154741 | C |  | 10 | 34.6 | [0, 0, 0, 10] | CT | 1 | 5.95E-05 | *rps*3 |
| mitochondrion | 263971 | C |  | 10 | 37 | [0, 0, 0, 10] | CT | 1 | 5.95E-05 | *rps*3 |
| mitochondrion | 98465 | C |  | 10 | 37 | [0, 0, 0, 10] | CT | 1 | 5.95E-05 | *atp*9 |

**Table S13 PTUs identified in organelle genomes of *S. divaricata.***

| Type | Transcript | Genes Covered by PTU |
| --- | --- | --- |
| PTU1_mitochondria | TRINITY_DN272_c0_g1_i1 | *rpl*5, *rps*14 |
| PTU2_mitochondria | TRINITY_DN298_c6_g12_i1 | *cox*1, *rps*7, *nad*4 |
| PTU1_chloroplast | TRINITY_DN226_c0_g1_i1 | *psa*A*, psa*B |
| PTU2_chloroplast | TRINITY_DN228_c0_g1_i1 | *clp*P,*rps*12 |
| PTU3_chloroplast | TRINITY_DN266_c0_g2_i1 | *mat*K,*trn*K-UUU |
| PTU4_chloroplast | TRINITY_DN278_c0_g1_i1 | *pet*A, *cem*A |
| PTU5_chloroplast | TRINITY_DN278_c1_g1_i1 | *ycf*4, *cem*A |
| PTU6_chloroplast | TRINITY_DN278_c1_g2_i1 | *pet*A, *acc*D, *cem*A, *psa*I |
| PTU7_chloroplast | TRINITY_DN287_c0_g3_i2 | *ndh*B, *rps*7 |
| PTU8_chloroplast | TRINITY_DN305_c0_g1_i1 | *psb*C, *psb*D |

**The transcripts were provided in the File S1.**

**Table S14. The MTPT fragments in the Apiales species.**

| cpgenome | mitogenome | Identities | length | cp-start | cp-end | mito-start | mito-end |
| --- | --- | --- | --- | --- | --- | --- | --- |
| *Saposhnikovia divaricata* | *Saposhnikovia divaricata* | 99.971 | 6813 | 101995 | 108807 | 119569 | 112758 |
| *Saposhnikovia divaricata* | *Saposhnikovia divaricata* | 99.971 | 6813 | 132228 | 139040 | 112758 | 119569 |
| *Saposhnikovia divaricata* | *Saposhnikovia divaricata* | 99.971 | 6813 | 101995 | 108807 | 150885 | 144074 |
| *Saposhnikovia divaricata* | *Saposhnikovia divaricata* | 99.971 | 6813 | 132228 | 139040 | 144074 | 150885 |
| *Saposhnikovia divaricata* | *Saposhnikovia divaricata* | 97.137 | 524 | 38935 | 39456 | 263233 | 262718 |
| *Saposhnikovia divaricata* | *Saposhnikovia divaricata* | 84.433 | 379 | 66613 | 66985 | 28759 | 28389 |
| *Saposhnikovia divaricata* | *Saposhnikovia divaricata* | 93.458 | 214 | 25512 | 25725 | 215088 | 214875 |
| *Saposhnikovia divaricata* | *Saposhnikovia divaricata* | 74.099 | 888 | 139020 | 139883 | 71079 | 70221 |
| *Saposhnikovia divaricata* | *Saposhnikovia divaricata* | 74.099 | 888 | 101152 | 102015 | 70221 | 71079 |
| *Saposhnikovia divaricata* | *Saposhnikovia divaricata* | 74.099 | 888 | 139020 | 139883 | 110682 | 109824 |
| *Saposhnikovia divaricata* | *Saposhnikovia divaricata* | 74.099 | 888 | 101152 | 102015 | 109824 | 110682 |
| *Saposhnikovia divaricata* | *Saposhnikovia divaricata* | 74.099 | 888 | 139020 | 139883 | 141998 | 141140 |
| *Saposhnikovia divaricata* | *Saposhnikovia divaricata* | 74.099 | 888 | 101152 | 102015 | 141140 | 141998 |
| *Saposhnikovia divaricata* | *Saposhnikovia divaricata* | 88.304 | 171 | 30970 | 31135 | 54311 | 54481 |
| *Saposhnikovia divaricata* | *Saposhnikovia divaricata* | 98.077 | 104 | 108704 | 108807 | 73257 | 73155 |
| *Saposhnikovia divaricata* | *Saposhnikovia divaricata* | 98.077 | 104 | 132228 | 132331 | 73155 | 73257 |
| *Saposhnikovia divaricata* | *Saposhnikovia divaricata* | 98.78 | 82 | 109185 | 109266 | 34749 | 34668 |
| *Saposhnikovia divaricata* | *Saposhnikovia divaricata* | 98.78 | 82 | 131769 | 131850 | 34668 | 34749 |
| *Saposhnikovia divaricata* | *Saposhnikovia divaricata* | 100 | 79 | 55 | 133 | 228770 | 228692 |
| *Saposhnikovia divaricata* | *Saposhnikovia divaricata* | 91.026 | 78 | 86835 | 86909 | 246004 | 246081 |
| *Bupleurum falcatum* | *Bupleurum falcatum* | 93.065 | 1716 | 103673 | 105365 | 340670 | 338979 |
| *Bupleurum falcatum* | *Bupleurum falcatum* | 93.065 | 1716 | 136537 | 138229 | 338979 | 340670 |
| *Bupleurum falcatum* | *Bupleurum falcatum* | 91.973 | 897 | 105052 | 105941 | 123536 | 122658 |
| *Bupleurum falcatum* | *Bupleurum falcatum* | 91.973 | 897 | 135961 | 136850 | 122658 | 123536 |
| *Bupleurum falcatum* | *Bupleurum falcatum* | 74.186 | 891 | 139125 | 139988 | 42639 | 41781 |
| *Bupleurum falcatum* | *Bupleurum falcatum* | 74.186 | 891 | 101914 | 102777 | 41781 | 42639 |
| *Bupleurum falcatum* | *Bupleurum falcatum* | 96.703 | 91 | 68021 | 68111 | 39677 | 39587 |
| *Bupleurum falcatum* | *Bupleurum falcatum* | 97.5 | 80 | 23 | 102 | 293990 | 293911 |
| *Bupleurum falcatum* | *Bupleurum falcatum* | 94.444 | 90 | 131908 | 131994 | 220869 | 220780 |
| *Bupleurum falcatum* | *Bupleurum falcatum* | 94.444 | 90 | 109908 | 109994 | 220780 | 220869 |
| *Bupleurum falcatum* | *Bupleurum falcatum* | 95.181 | 83 | 31739 | 31821 | 399019 | 399101 |
| *Bupleurum falcatum* | *Bupleurum falcatum* | 93.671 | 79 | 68018 | 68096 | 72858 | 72936 |
| *Bupleurum falcatum* | *Bupleurum falcatum* | 93.243 | 74 | 53949 | 54022 | 297326 | 297253 |
| *Bupleurum falcatum* | *Bupleurum falcatum* | 89.189 | 74 | 123047 | 123119 | 162762 | 162834 |
| *Bupleurum falcatum* | *Bupleurum falcatum* | 89.189 | 74 | 123047 | 123119 | 444943 | 444871 |
| *Bupleurum falcatum* | *Bupleurum falcatum* | 93.478 | 46 | 123577 | 123622 | 162840 | 162885 |
| *Bupleurum falcatum* | *Bupleurum falcatum* | 93.478 | 46 | 123577 | 123622 | 444865 | 444820 |
| *Bupleurum falcatum* | *Bupleurum falcatum* | 97.222 | 36 | 102640 | 102675 | 70266 | 70231 |
| *Bupleurum falcatum* | *Bupleurum falcatum* | 97.222 | 36 | 139227 | 139262 | 70231 | 70266 |
| *Daucus carota* | *Daucus carota* | 99.456 | 2389 | 153528 | 155911 | 45518 | 47902 |
| *Daucus carota* | *Daucus carota* | 99.456 | 2388 | 84244 | 86626 | 47901 | 45518 |
| *Daucus carota* | *Daucus carota* | 98.856 | 1311 | 139415 | 140717 | 103321 | 102017 |
| *Daucus carota* | *Daucus carota* | 98.856 | 1311 | 99437 | 100739 | 102017 | 103321 |
| *Daucus carota* | *Daucus carota* | 98.635 | 1026 | 40570 | 41582 | 232562 | 231537 |
| *Daucus carota* | *Daucus carota* | 98.635 | 1026 | 40570 | 41582 | 279944 | 278919 |
| *Daucus carota* | *Daucus carota* | 97.164 | 529 | 39924 | 40447 | 233088 | 232560 |
| *Daucus carota* | *Daucus carota* | 97.164 | 529 | 39924 | 40447 | 280470 | 279942 |
| *Daucus carota* | *Daucus carota* | 96.181 | 288 | 37576 | 37856 | 127383 | 127670 |
| *Daucus carota* | *Daucus carota* | 96.935 | 261 | 37878 | 38138 | 127658 | 127918 |
| *Daucus carota* | *Daucus carota* | 95.673 | 208 | 25177 | 25384 | 13906 | 13699 |
| *Daucus carota* | *Daucus carota* | 83.113 | 379 | 66239 | 66611 | 3779 | 3409 |
| *Daucus carota* | *Daucus carota* | 73.761 | 888 | 101261 | 102124 | 27009 | 26151 |
| *Daucus carota* | *Daucus carota* | 73.761 | 888 | 138030 | 138893 | 26151 | 27009 |
| *Daucus carota* | *Daucus carota* | 100 | 123 | 144911 | 145033 | 22573 | 22451 |
| *Daucus carota* | *Daucus carota* | 100 | 123 | 95121 | 95243 | 22451 | 22573 |
| *Daucus carota* | *Daucus carota* | 94.017 | 117 | 30572 | 30687 | 18417 | 18301 |
| *Daucus carota* | *Daucus carota* | 98.734 | 79 | 1 | 79 | 47903 | 47981 |
| *Daucus carota* | *Daucus carota* | 100 | 72 | 140719 | 140790 | 190737 | 190666 |
| *Daucus carota* | *Daucus carota* | 100 | 72 | 99364 | 99435 | 190666 | 190737 |
| *Daucus carota* | *Daucus carota* | 95.181 | 83 | 109196 | 109277 | 173138 | 173056 |
| *Daucus carota* | *Daucus carota* | 95.181 | 83 | 130877 | 130958 | 173056 | 173138 |
| *Daucus carota* | *Daucus carota* | 97.297 | 74 | 140783 | 140856 | 108211 | 108138 |
| *Daucus carota* | *Daucus carota* | 97.297 | 74 | 99298 | 99371 | 108138 | 108211 |
| *Daucus carota* | *Daucus carota* | 97.059 | 68 | 103064 | 103131 | 81178 | 81111 |
| *Daucus carota* | *Daucus carota* | 97.059 | 68 | 137023 | 137090 | 81111 | 81178 |
| *Daucus carota* | *Daucus carota* | 95.714 | 70 | 38506 | 38575 | 21670 | 21738 |
| *Daucus carota* | *Daucus carota* | 100 | 46 | 139053 | 139098 | 180418 | 180373 |
| *Daucus carota* | *Daucus carota* | 100 | 46 | 101056 | 101101 | 180373 | 180418 |
| *Daucus carota* | *Daucus carota* | 95.918 | 49 | 150030 | 150078 | 188948 | 188900 |
| *Daucus carota* | *Daucus carota* | 95.918 | 49 | 90076 | 90124 | 188900 | 188948 |
| *Diplostephium hartwegii* | *Diplostephium hartwegii* | 77.608 | 1112 | 65395 | 66446 | 205815 | 206892 |
| *Diplostephium hartwegii* | *Diplostephium hartwegii* | 93.385 | 257 | 144112 | 144368 | 200074 | 199827 |
| *Diplostephium hartwegii* | *Diplostephium hartwegii* | 93.385 | 257 | 91456 | 91712 | 199827 | 200074 |
| *Diplostephium hartwegii* | *Diplostephium hartwegii* | 73.761 | 888 | 100199 | 101062 | 110174 | 109316 |
| *Diplostephium hartwegii* | *Diplostephium hartwegii* | 73.761 | 888 | 134762 | 135625 | 109316 | 110174 |
| *Diplostephium hartwegii* | *Diplostephium hartwegii* | 99.306 | 144 | 100330 | 100473 | 144 | 1 |
| *Diplostephium hartwegii* | *Diplostephium hartwegii* | 99.306 | 144 | 135351 | 135494 | 1 | 144 |
| *Diplostephium hartwegii* | *Diplostephium hartwegii* | 94.118 | 136 | 35158 | 35292 | 59023 | 59153 |
| *Diplostephium hartwegii* | *Diplostephium hartwegii* | 89.286 | 140 | 11138 | 11274 | 6892 | 6753 |
| *Diplostephium hartwegii* | *Diplostephium hartwegii* | 98.947 | 95 | 105179 | 105273 | 277718 | 277624 |
| *Diplostephium hartwegii* | *Diplostephium hartwegii* | 98.947 | 95 | 130551 | 130645 | 277624 | 277718 |
| *Diplostephium hartwegii* | *Diplostephium hartwegii* | 97.5 | 80 | 2 | 81 | 134882 | 134961 |
| *Diplostephium hartwegii* | *Diplostephium hartwegii* | 92.941 | 85 | 127861 | 127945 | 80499 | 80415 |
| *Diplostephium hartwegii* | *Diplostephium hartwegii* | 92.941 | 85 | 107879 | 107963 | 80415 | 80499 |
| *Diplostephium hartwegii* | *Diplostephium hartwegii* | 94.937 | 79 | 51847 | 51925 | 254298 | 254220 |
| *Chrysanthemum boreale* | *Chrysanthemum boreale* | 98.122 | 2556 | 36696 | 39250 | 180697 | 178150 |
| *Chrysanthemum boreale* | *Chrysanthemum boreale* | 78.128 | 1111 | 64490 | 65560 | 161190 | 160134 |
| *Chrysanthemum boreale* | *Chrysanthemum boreale* | 82.572 | 591 | 103427 | 104014 | 22636 | 22088 |
| *Chrysanthemum boreale* | *Chrysanthemum boreale* | 82.572 | 591 | 129816 | 130403 | 22088 | 22636 |
| *Chrysanthemum boreale* | *Chrysanthemum boreale* | 93.156 | 263 | 90429 | 90691 | 202768 | 202515 |
| *Chrysanthemum boreale* | *Chrysanthemum boreale* | 93.156 | 263 | 143139 | 143401 | 202515 | 202768 |
| *Chrysanthemum boreale* | *Chrysanthemum boreale* | 73.761 | 888 | 99125 | 99988 | 198466 | 197608 |
| *Chrysanthemum boreale* | *Chrysanthemum boreale* | 73.761 | 888 | 133842 | 134705 | 197608 | 198466 |
| *Chrysanthemum boreale* | *Chrysanthemum boreale* | 81.919 | 271 | 104005 | 104274 | 22008 | 21771 |
| *Chrysanthemum boreale* | *Chrysanthemum boreale* | 81.919 | 271 | 129556 | 129825 | 21771 | 22008 |
| *Chrysanthemum boreale* | *Chrysanthemum boreale* | 97.5 | 80 | 8 | 87 | 68146 | 68225 |
| *Chrysanthemum boreale* | *Chrysanthemum boreale* | 96.429 | 84 | 106816 | 106898 | 171987 | 171904 |
| *Chrysanthemum boreale* | *Chrysanthemum boreale* | 96.429 | 84 | 126932 | 127014 | 171904 | 171987 |
| *Chrysanthemum boreale* | *Chrysanthemum boreale* | 97.468 | 79 | 51878 | 51956 | 134611 | 134689 |
| *Helianthus annuus* | *Helianthus annuus* | 99.083 | 1199 | 39544 | 40741 | 212274 | 211076 |
| *Helianthus annuus* | *Helianthus annuus* | 98.901 | 819 | 101689 | 102506 | 293750 | 292932 |
| *Helianthus annuus* | *Helianthus annuus* | 98.901 | 819 | 132129 | 132946 | 292932 | 293750 |
| *Helianthus annuus* | *Helianthus annuus* | 98.779 | 819 | 101689 | 102506 | 57420 | 56602 |
| *Helianthus annuus* | *Helianthus annuus* | 98.779 | 819 | 132129 | 132946 | 56602 | 57420 |
| *Helianthus annuus* | *Helianthus annuus* | 99.52 | 417 | 98827 | 99243 | 39344 | 38928 |
| *Helianthus annuus* | *Helianthus annuus* | 99.52 | 417 | 135392 | 135808 | 38928 | 39344 |
| *Helianthus annuus* | *Helianthus annuus* | 80.164 | 731 | 65474 | 66179 | 262175 | 261479 |
| *Helianthus annuus* | *Helianthus annuus* | 73.761 | 888 | 99437 | 100300 | 141850 | 140992 |
| *Helianthus annuus* | *Helianthus annuus* | 73.761 | 888 | 134335 | 135198 | 140992 | 141850 |
| *Helianthus annuus* | *Helianthus annuus* | 90.968 | 155 | 38130 | 38283 | 183677 | 183826 |
| *Helianthus annuus* | *Helianthus annuus* | 90.678 | 118 | 104508 | 104625 | 56161 | 56053 |
| *Helianthus annuus* | *Helianthus annuus* | 90.678 | 118 | 130010 | 130127 | 56053 | 56161 |
| *Helianthus annuus* | *Helianthus annuus* | 90.678 | 118 | 104508 | 104625 | 292491 | 292383 |
| *Helianthus annuus* | *Helianthus annuus* | 90.678 | 118 | 130010 | 130127 | 292383 | 292491 |
| *Helianthus annuus* | *Helianthus annuus* | 79.909 | 219 | 16999 | 17201 | 285947 | 285729 |
| *Helianthus annuus* | *Helianthus annuus* | 86.567 | 134 | 11598 | 11728 | 51680 | 51550 |
| *Helianthus annuus* | *Helianthus annuus* | 97.436 | 78 | 1 | 78 | 87833 | 87910 |
| *Helianthus annuus* | *Helianthus annuus* | 95.238 | 84 | 127368 | 127450 | 6666 | 6583 |
| *Helianthus annuus* | *Helianthus annuus* | 95.238 | 84 | 107185 | 107267 | 6583 | 6666 |
| *Helianthus annuus* | *Helianthus annuus* | 94.937 | 79 | 51617 | 51695 | 118553 | 118631 |
| *Helianthus annuus* | *Helianthus annuus* | 100 | 28 | 52347 | 52374 | 65925 | 65952 |
| *Helianthus strumosus* | *Helianthus strumosus* | 99.898 | 2936 | 129986 | 132921 | 202567 | 199633 |
| *Helianthus strumosus* | *Helianthus strumosus* | 99.898 | 2936 | 101690 | 104625 | 199633 | 202567 |
| *Helianthus strumosus* | *Helianthus strumosus* | 99.668 | 1204 | 39588 | 40791 | 61358 | 62561 |
| *Helianthus strumosus* | *Helianthus strumosus* | 99.76 | 417 | 135368 | 135784 | 219689 | 219273 |
| *Helianthus strumosus* | *Helianthus strumosus* | 99.76 | 417 | 98827 | 99243 | 219273 | 219689 |
| *Helianthus strumosus* | *Helianthus strumosus* | 80.966 | 725 | 65545 | 66245 | 187753 | 187057 |
| *Helianthus strumosus* | *Helianthus strumosus* | 73.761 | 888 | 134311 | 135174 | 130607 | 129749 |
| *Helianthus strumosus* | *Helianthus strumosus* | 73.761 | 888 | 99437 | 100300 | 129749 | 130607 |
| *Helianthus strumosus* | *Helianthus strumosus* | 90.968 | 155 | 38180 | 38333 | 228475 | 228326 |
| *Helianthus strumosus* | *Helianthus strumosus* | 89.683 | 126 | 11621 | 11743 | 206943 | 207068 |
| *Helianthus strumosus* | *Helianthus strumosus* | 79.909 | 219 | 17041 | 17243 | 72823 | 72605 |
| *Helianthus strumosus* | *Helianthus strumosus* | 79.909 | 219 | 17041 | 17243 | 272783 | 272565 |
| *Helianthus strumosus* | *Helianthus strumosus* | 97.436 | 78 | 1 | 78 | 15649 | 15726 |
| *Helianthus strumosus* | *Helianthus strumosus* | 95.238 | 84 | 127346 | 127428 | 80214 | 80131 |
| *Helianthus strumosus* | *Helianthus strumosus* | 95.238 | 84 | 107183 | 107265 | 80131 | 80214 |
| *Helianthus strumosus* | *Helianthus strumosus* | 94.937 | 79 | 51657 | 51735 | 46336 | 46414 |
| *Helianthus strumosus* | *Helianthus strumosus* | 100 | 28 | 52389 | 52416 | 274798 | 274825 |
| *Helianthus grosseserratus* | *Helianthus grosseserratus* | 99.165 | 1198 | 39597 | 40794 | 103190 | 101994 |
| *Helianthus grosseserratus* | *Helianthus grosseserratus* | 99.76 | 417 | 135341 | 135757 | 240015 | 239599 |
| *Helianthus grosseserratus* | *Helianthus grosseserratus* | 99.76 | 417 | 98820 | 99236 | 239599 | 240015 |
| *Helianthus grosseserratus* | *Helianthus grosseserratus* | 80.966 | 725 | 65558 | 66258 | 178664 | 177968 |
| *Helianthus grosseserratus* | *Helianthus grosseserratus* | 73.761 | 888 | 99430 | 100293 | 68688 | 67830 |
| *Helianthus grosseserratus* | *Helianthus grosseserratus* | 73.761 | 888 | 134284 | 135147 | 67830 | 68688 |
| *Helianthus grosseserratus* | *Helianthus grosseserratus* | 90.968 | 155 | 38183 | 38336 | 249482 | 249333 |
| *Helianthus grosseserratus* | *Helianthus grosseserratus* | 89.683 | 126 | 11625 | 11747 | 227268 | 227393 |
| *Helianthus grosseserratus* | *Helianthus grosseserratus* | 90.083 | 121 | 129959 | 130079 | 125784 | 125673 |
| *Helianthus grosseserratus* | *Helianthus grosseserratus* | 90.083 | 121 | 104498 | 104618 | 125673 | 125784 |
| *Helianthus grosseserratus* | *Helianthus grosseserratus* | 90.083 | 121 | 129959 | 130079 | 222892 | 222781 |
| *Helianthus grosseserratus* | *Helianthus grosseserratus* | 90.083 | 121 | 104498 | 104618 | 222781 | 222892 |
| *Helianthus grosseserratus* | *Helianthus grosseserratus* | 79.909 | 219 | 17045 | 17247 | 132220 | 132438 |
| *Helianthus grosseserratus* | *Helianthus grosseserratus* | 97.436 | 78 | 1 | 78 | 15701 | 15778 |
| *Helianthus grosseserratus* | *Helianthus grosseserratus* | 95.238 | 84 | 107176 | 107258 | 211220 | 211137 |
| *Helianthus grosseserratus* | *Helianthus grosseserratus* | 95.238 | 84 | 127319 | 127401 | 211137 | 211220 |
| *Helianthus grosseserratus* | *Helianthus grosseserratus* | 94.937 | 79 | 51661 | 51739 | 45488 | 45566 |
| *Helianthus grosseserratus* | *Helianthus grosseserratus* | 100 | 28 | 52393 | 52420 | 119421 | 119394 |
| *Platycodon grandiflorus* | *Platycodon grandiflorus* | 87.912 | 819 | 69744 | 70543 | 755074 | 754283 |
| *Platycodon grandiflorus* | *Platycodon grandiflorus* | 95.27 | 444 | 47706 | 48149 | 1069667 | 1070110 |
| *Platycodon grandiflorus* | *Platycodon grandiflorus* | 99.133 | 346 | 93102 | 93447 | 890973 | 890628 |
| *Platycodon grandiflorus* | *Platycodon grandiflorus* | 99.133 | 346 | 157484 | 157829 | 890628 | 890973 |
| *Platycodon grandiflorus* | *Platycodon grandiflorus* | 90.583 | 223 | 145844 | 146066 | 5436 | 5219 |
| *Platycodon grandiflorus* | *Platycodon grandiflorus* | 90.583 | 223 | 104865 | 105087 | 5219 | 5436 |
| *Platycodon grandiflorus* | *Platycodon grandiflorus* | 85.467 | 289 | 52079 | 52367 | 470188 | 470458 |
| *Platycodon grandiflorus* | *Platycodon grandiflorus* | 80.353 | 397 | 158843 | 159220 | 379063 | 378667 |
| *Platycodon grandiflorus* | *Platycodon grandiflorus* | 80.353 | 397 | 91711 | 92088 | 378667 | 379063 |
| *Platycodon grandiflorus* | *Platycodon grandiflorus* | 85.169 | 236 | 72116 | 72351 | 32382 | 32605 |
| *Platycodon grandiflorus* | *Platycodon grandiflorus* | 95 | 140 | 48564 | 48703 | 697008 | 697147 |
| *Platycodon grandiflorus* | *Platycodon grandiflorus* | 94.964 | 139 | 102952 | 103090 | 926669 | 926531 |
| *Platycodon grandiflorus* | *Platycodon grandiflorus* | 94.964 | 139 | 147841 | 147979 | 926531 | 926669 |
| *Platycodon grandiflorus* | *Platycodon grandiflorus* | 80.783 | 281 | 156684 | 156958 | 1025062 | 1024793 |
| *Platycodon grandiflorus* | *Platycodon grandiflorus* | 80.783 | 281 | 93973 | 94247 | 1024793 | 1025062 |
| *Platycodon grandiflorus* | *Platycodon grandiflorus* | 80.669 | 269 | 149539 | 149801 | 164369 | 164109 |
| *Platycodon grandiflorus* | *Platycodon grandiflorus* | 80.669 | 269 | 101130 | 101392 | 164109 | 164369 |
| *Platycodon grandiflorus* | *Platycodon grandiflorus* | 80.669 | 269 | 149539 | 149801 | 1234800 | 1234540 |
| *Platycodon grandiflorus* | *Platycodon grandiflorus* | 80.669 | 269 | 101130 | 101392 | 1234540 | 1234800 |
| *Platycodon grandiflorus* | *Platycodon grandiflorus* | 91.15 | 113 | 28663 | 28775 | 805462 | 805350 |
| *Platycodon grandiflorus* | *Platycodon grandiflorus* | 75.484 | 310 | 150087 | 150390 | 163833 | 163528 |
| *Platycodon grandiflorus* | *Platycodon grandiflorus* | 75.484 | 310 | 100541 | 100844 | 163528 | 163833 |
| *Platycodon grandiflorus* | *Platycodon grandiflorus* | 75.484 | 310 | 150087 | 150390 | 1234264 | 1233959 |
| *Platycodon grandiflorus* | *Platycodon grandiflorus* | 75.484 | 310 | 100541 | 100844 | 1233959 | 1234264 |
| *Platycodon grandiflorus* | *Platycodon grandiflorus* | 96.386 | 83 | 30222 | 30304 | 181855 | 181773 |
| *Platycodon grandiflorus* | *Platycodon grandiflorus* | 97.5 | 80 | 8 | 86 | 567646 | 567725 |
| *Platycodon grandiflorus* | *Platycodon grandiflorus* | 84.564 | 149 | 58086 | 58222 | 1018306 | 1018159 |
| *Platycodon grandiflorus* | *Platycodon grandiflorus* | 94.872 | 78 | 50793 | 50870 | 697573 | 697650 |
| *Platycodon grandiflorus* | *Platycodon grandiflorus* | 96.923 | 65 | 152254 | 152318 | 901295 | 901231 |
| *Platycodon grandiflorus* | *Platycodon grandiflorus* | 96.923 | 65 | 98613 | 98677 | 901231 | 901295 |
| *Platycodon grandiflorus* | *Platycodon grandiflorus* | 98.333 | 60 | 16009 | 16068 | 499347 | 499406 |
| *Platycodon grandiflorus* | *Platycodon grandiflorus* | 73.477 | 279 | 7800 | 8057 | 254739 | 254474 |
| *Codonopsis lanceolata* | *Codonopsis lanceolata* | 99.032 | 2995 | 95055 | 98049 | 403609 | 400628 |
| *Codonopsis lanceolata* | *Codonopsis lanceolata* | 99.032 | 2995 | 157268 | 160262 | 400628 | 403609 |
| *Codonopsis lanceolata* | *Codonopsis lanceolata* | 99.681 | 1566 | 89498 | 91063 | 27452 | 25887 |
| *Codonopsis lanceolata* | *Codonopsis lanceolata* | 99.681 | 1566 | 164254 | 165819 | 25887 | 27452 |
| *Codonopsis lanceolata* | *Codonopsis lanceolata* | 95.038 | 262 | 45042 | 45302 | 284951 | 285210 |
| *Codonopsis lanceolata* | *Codonopsis lanceolata* | 84.488 | 303 | 25844 | 26135 | 76392 | 76099 |
| *Codonopsis lanceolata* | *Codonopsis lanceolata* | 75.847 | 472 | 152682 | 153142 | 84806 | 84354 |
| *Codonopsis lanceolata* | *Codonopsis lanceolata* | 75.847 | 472 | 102175 | 102635 | 84354 | 84806 |
| *Codonopsis lanceolata* | *Codonopsis lanceolata* | 90.164 | 122 | 69015 | 69135 | 215210 | 215089 |
| *Codonopsis lanceolata* | *Codonopsis lanceolata* | 95.455 | 88 | 23316 | 23403 | 137907 | 137820 |
| *Codonopsis lanceolata* | *Codonopsis lanceolata* | 95 | 80 | 2 | 80 | 79619 | 79540 |
| *Codonopsis lanceolata* | *Codonopsis lanceolata* | 74.598 | 311 | 153236 | 153539 | 84263 | 83959 |
| *Codonopsis lanceolata* | *Codonopsis lanceolata* | 74.598 | 311 | 101778 | 102081 | 83959 | 84263 |
| *Codonopsis lanceolata* | *Codonopsis lanceolata* | 91.667 | 84 | 109457 | 109539 | 301150 | 301067 |
| *Codonopsis lanceolata* | *Codonopsis lanceolata* | 91.667 | 84 | 145778 | 145860 | 301067 | 301150 |
| *Codonopsis lanceolata* | *Codonopsis lanceolata* | 92 | 75 | 47382 | 47455 | 135171 | 135245 |
| *Codonopsis lanceolata* | *Codonopsis lanceolata* | 90.667 | 75 | 21847 | 21920 | 135245 | 135171 |
| *Codonopsis lanceolata* | *Codonopsis lanceolata* | 95 | 60 | 90700 | 90759 | 26202 | 26143 |
| *Codonopsis lanceolata* | *Codonopsis lanceolata* | 95 | 60 | 164558 | 164617 | 26143 | 26202 |
| *Codonopsis lanceolata* | *Codonopsis lanceolata* | 95 | 60 | 90748 | 90807 | 26250 | 26191 |
| *Codonopsis lanceolata* | *Codonopsis lanceolata* | 95 | 60 | 164510 | 164569 | 26191 | 26250 |
| *Ilex pubescens* | *Ilex pubescens* | 99.936 | 1564 | 148307 | 149869 | 348778 | 347215 |
| *Ilex pubescens* | *Ilex pubescens* | 99.936 | 1564 | 95016 | 96578 | 347215 | 348778 |
| *Ilex pubescens* | *Ilex pubescens* | 99.345 | 1375 | 72751 | 74123 | 342072 | 340698 |
| *Ilex pubescens* | *Ilex pubescens* | 87.243 | 972 | 83716 | 84658 | 500391 | 499435 |
| *Ilex pubescens* | *Ilex pubescens* | 88.832 | 394 | 69169 | 69553 | 43059 | 43443 |
| *Ilex pubescens* | *Ilex pubescens* | 80.5 | 600 | 50981 | 51565 | 328598 | 328026 |
| *Ilex pubescens* | *Ilex pubescens* | 99.563 | 229 | 147196 | 147424 | 348996 | 348768 |
| *Ilex pubescens* | *Ilex pubescens* | 99.563 | 229 | 97461 | 97689 | 348768 | 348996 |
| *Ilex pubescens* | *Ilex pubescens* | 83.871 | 434 | 84746 | 85165 | 499278 | 498853 |
| *Ilex pubescens* | *Ilex pubescens* | 73.986 | 888 | 103773 | 104636 | 39511 | 38653 |
| *Ilex pubescens* | *Ilex pubescens* | 73.986 | 888 | 140249 | 141112 | 38653 | 39511 |
| *Ilex pubescens* | *Ilex pubescens* | 95 | 180 | 58546 | 58725 | 136664 | 136490 |
| *Ilex pubescens* | *Ilex pubescens* | 99.32 | 147 | 36720 | 36866 | 235708 | 235854 |
| *Ilex pubescens* | *Ilex pubescens* | 98.039 | 153 | 110671 | 110820 | 401269 | 401117 |
| *Ilex pubescens* | *Ilex pubescens* | 98.039 | 153 | 134065 | 134214 | 401117 | 401269 |
| *Ilex pubescens* | *Ilex pubescens* | 89.944 | 179 | 32024 | 32202 | 81311 | 81134 |
| *Ilex pubescens* | *Ilex pubescens* | 94.624 | 93 | 133036 | 133128 | 222398 | 222307 |
| *Ilex pubescens* | *Ilex pubescens* | 94.624 | 93 | 111757 | 111849 | 222307 | 222398 |
| *Ilex pubescens* | *Ilex pubescens* | 94.382 | 89 | 59 | 147 | 49494 | 49407 |
| *Ilex pubescens* | *Ilex pubescens* | 92.405 | 79 | 54679 | 54757 | 232854 | 232932 |
| *Ilex pubescens* | *Ilex pubescens* | 100 | 44 | 133073 | 133116 | 319430 | 319387 |
| *Ilex pubescens* | *Ilex pubescens* | 100 | 44 | 111769 | 111812 | 319387 | 319430 |
| *Ilex pubescens* | *Ilex pubescens* | 86.667 | 75 | 155659 | 155733 | 156058 | 155990 |
| *Ilex pubescens* | *Ilex pubescens* | 86.667 | 75 | 89152 | 89226 | 155990 | 156058 |
| *Ilex pubescens* | *Ilex pubescens* | 97.778 | 45 | 40450 | 40494 | 397405 | 397361 |
| *Ilex pubescens* | *Ilex pubescens* | 97.561 | 41 | 106377 | 106417 | 206134 | 206094 |
| *Ilex pubescens* | *Ilex pubescens* | 97.561 | 41 | 138468 | 138508 | 206094 | 206134 |

*Saposhnikovia divaricata*: MZ089852.1,MZ128146.1; *Bupleurum falcatum*: NC_027834.1,NC_035962.1; *Daucus carota*: NC_008325.1,NC_017855.1; *Diplostephium hartwegii*: NC_034832.1,NC_034354.1; *Chrysanthemum boreale:* NC_037388.1,NC_039757.1; *Helianthus annuus:* NC_007977.1,NC_023337.1; *Helianthus strumosus:* NC_023113.1,NC_051990.1; *Helianthus grosseserratus:* NC_023108.1,NC_051989.1; *Platycodon grandiflorus:* NC_035624.1,NC_035958.1; *Codonopsis lanceolata:* MH251613.1,NC_037949.1; *Ilex pubescens:* KX426467.1,NC_045078.1;

**Table S15 The common MTPT DNA fragments in Apiales species.**

| cpgenome | mitogenome | Identities | length | cp-start | cp-end | mito-start | mito-end |
| --- | --- | --- | --- | --- | --- | --- | --- |
| *Saposhnikovia divaricata* | *Bupleurum falcatum:* | 74 | 891 | 1 | 864 | 42639 | 41781 |
| *Saposhnikovia divaricata* | *Saposhnikovia divaricata* | 74 | 888 | 1 | 864 | 71079 | 70221 |
| *Saposhnikovia divaricata* | *Saposhnikovia divaricata* | 74 | 888 | 1 | 864 | 110682 | 109824 |
| *Saposhnikovia divaricata* | *Saposhnikovia divaricata* | 74 | 888 | 1 | 864 | 141998 | 141140 |
| *Saposhnikovia divaricata* | *Ilex pubescens* | 73 | 888 | 1 | 864 | 38653 | 39511 |
| *Saposhnikovia divaricata* | *Daucus carota* | 73 | 888 | 1 | 864 | 26151 | 27009 |
| *Saposhnikovia divaricata* | *Helianthus grosseserratus* | 73 | 888 | 1 | 864 | 67830 | 68688 |
| *Saposhnikovia divaricata* | *Helianthus strumosus:* | 73 | 888 | 1 | 864 | 130607 | 129749 |
| *Saposhnikovia divaricata* | *Helianthus annuus* | 73 | 888 | 1 | 864 | 140992 | 141850 |
| *Saposhnikovia divaricata* | *Chrysanthemum boreale* | 73 | 888 | 1 | 864 | 197608 | 198466 |
| *Saposhnikovia divaricata* | *Diplostephium hartwegii* | 73 | 888 | 1 | 864 | 109316 | 110174 |
| *Saposhnikovia divaricata* | *Codonopsis lanceolata* | 76 | 471 | 1 | 461 | 84806 | 84354 |
| *Saposhnikovia divaricata* | *Codonopsis lanceolata* | 75 | 315 | 555 | 864 | 84263 | 83952 |
| *Saposhnikovia divaricata* | *Platycodon grandiflorus* | 75 | 465 | 7 | 461 | 164369 | 163924 |
| *Saposhnikovia divaricata* | *Platycodon grandiflorus* | 75 | 465 | 7 | 461 | 1234800 | 1234355 |
| *Saposhnikovia divaricata* | *Platycodon grandiflorus* | 76 | 315 | 555 | 864 | 163833 | 163522 |
| *Saposhnikovia divaricata* | *Platycodon grandiflorus* | 76 | 315 | 555 | 864 | 1234264 | 1233953 |

*Saposhnikovia divaricata*: MZ128146, MZ089852; *Bupleurum falcatum*: NC_035962; *Daucus carota*: NC_017855; *Diplostephium hartwegii*: NC_034354; *Chrysanthemum boreale*: NC_039757; *Helianthus annuus*: NC_023337; *Helianthus strumosus*: NC_051990; *Helianthus grosseserratus*: NC_051989; *Platycodon grandiflorus*: NC_035958; *Codonopsis lanceolata*: NC_037949; *Ilex pubescens*: NC_045078;

**Supplementary Figures:**

**
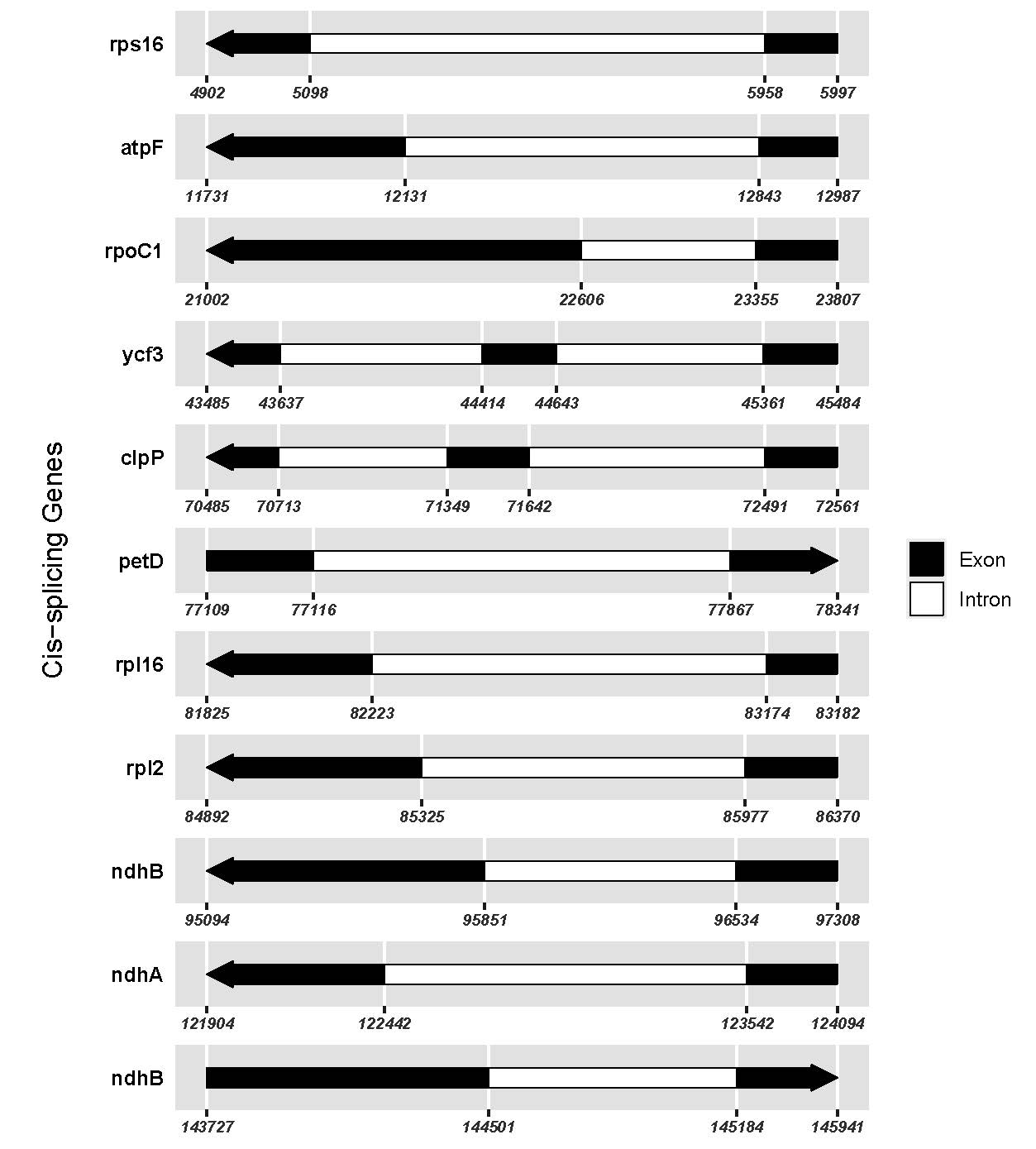
**

**Supplementary Figure 1. Cis-splicing gene map generated for the chloroplast genome of** ***Saposhnikovia divaricata*.** The genes are arranged from top to bottom based on their order on the chloroplast genome. The gene names are shown on the left, and the gene structures are shown on the right. The exons are shown in black; the introns are shown in white. The coordinates show the positions of the exons adjusted after the CSA algorithm. The arrow indicates the sense direction of the gene.

**
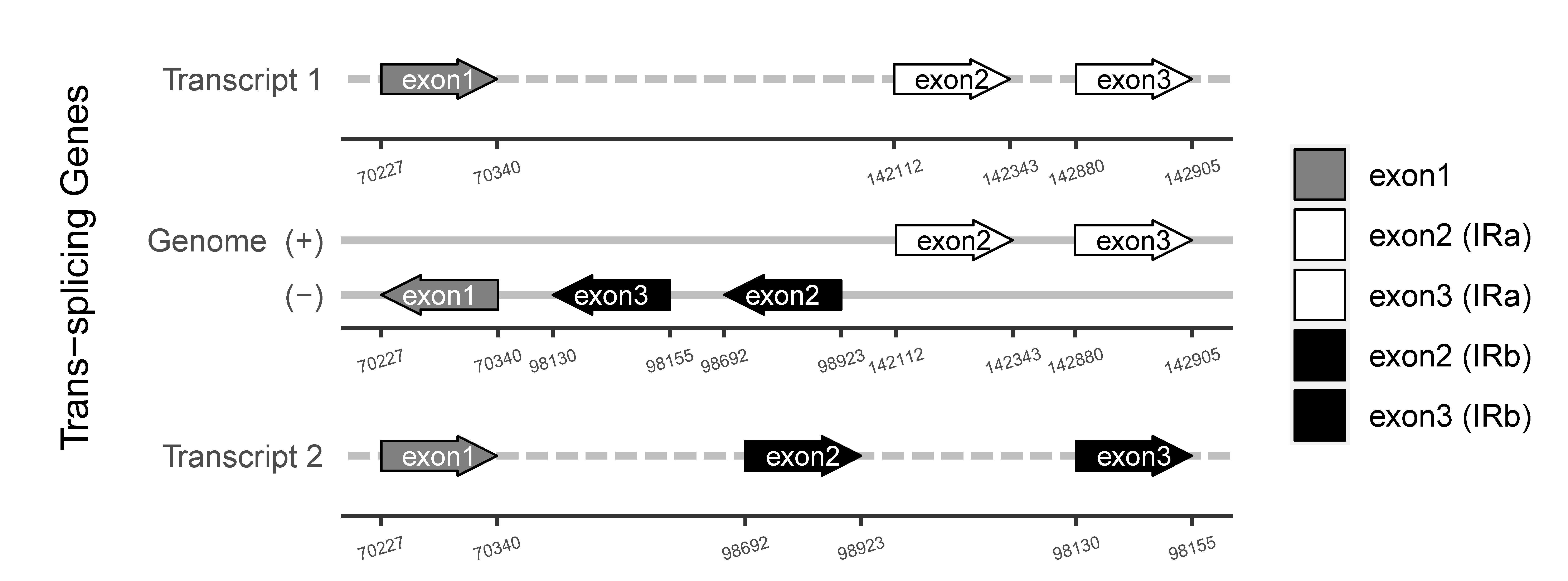
**

**Supplementary Figure 2.** Trans-splicing gene map generated for the chloroplast genome of *Arabidopsis thaliana*. Panels (A) and (B) show the graphs generated based on the three-exon model.


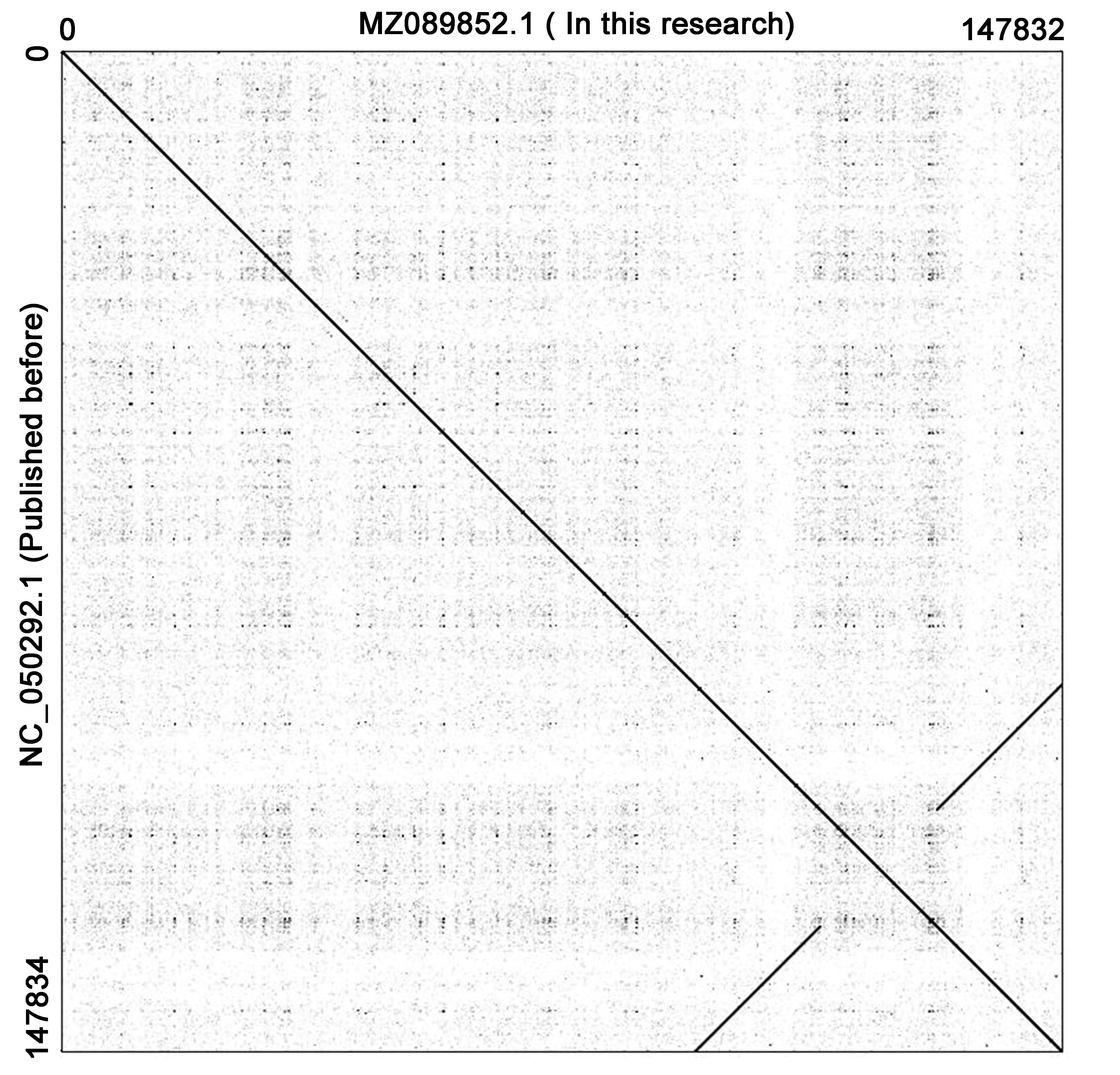


**Supplementary Figure 3. The dotplot of two *Saposhnikovia divaricata* chloroplast genomes.** The horizontal line represents the chloroplast genome assembled in this study (MZ089852.1) and the vertical line represents the one reported before (NC_050292.1). Their total lengths differ by two base pairs.


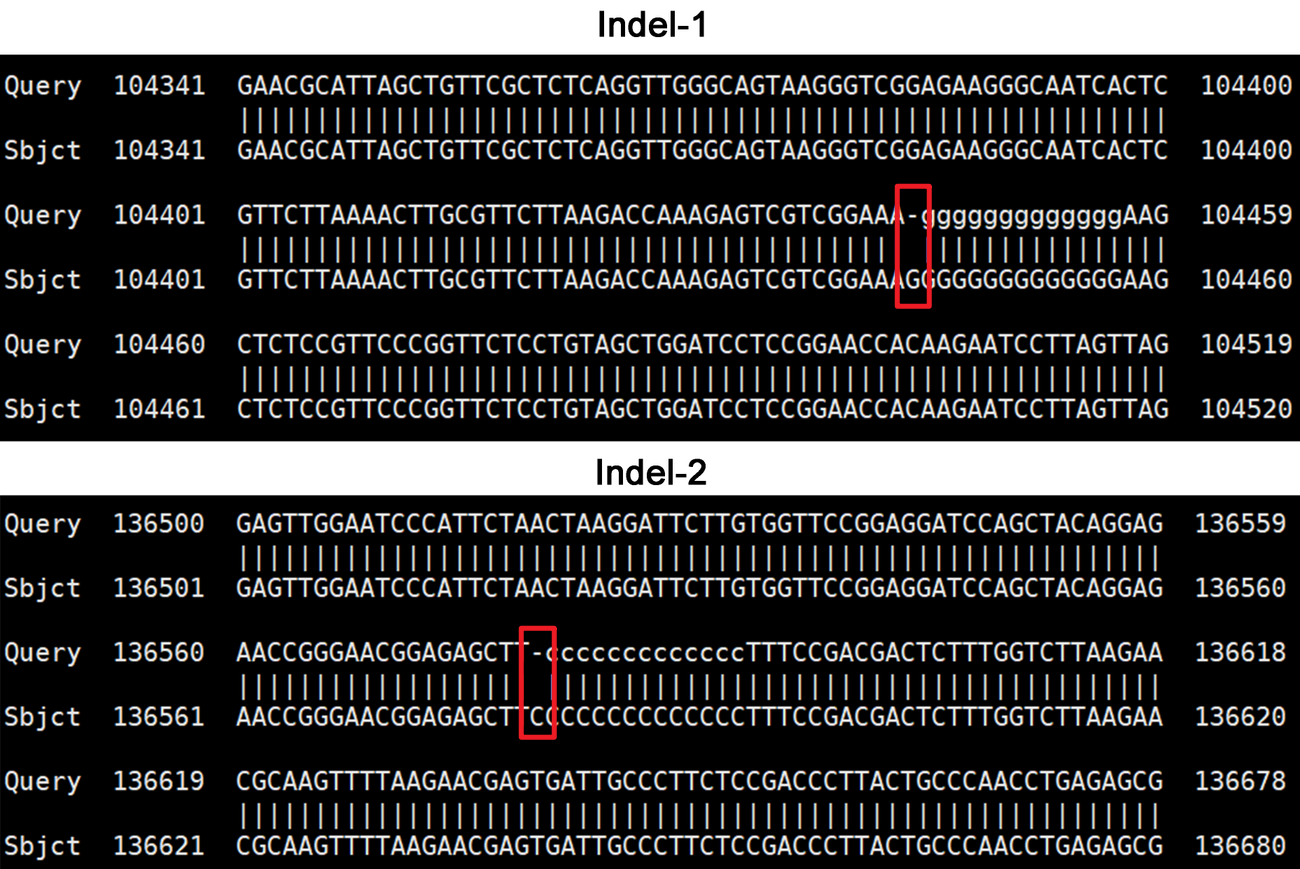


**Supplementary Figure 4.** The difference between the two chloroplast genomes identified by BLASTN. “Query” represents the chloroplast genome from this study (MZ089852.1). “Sbjct” represents the previously discovered chloroplast genome (NC_050292.1). There are two indels in total, each marked with a red rectangular frame. Both Indel-1 and Indel-2 are located in the introns of trnA-UGC genes.


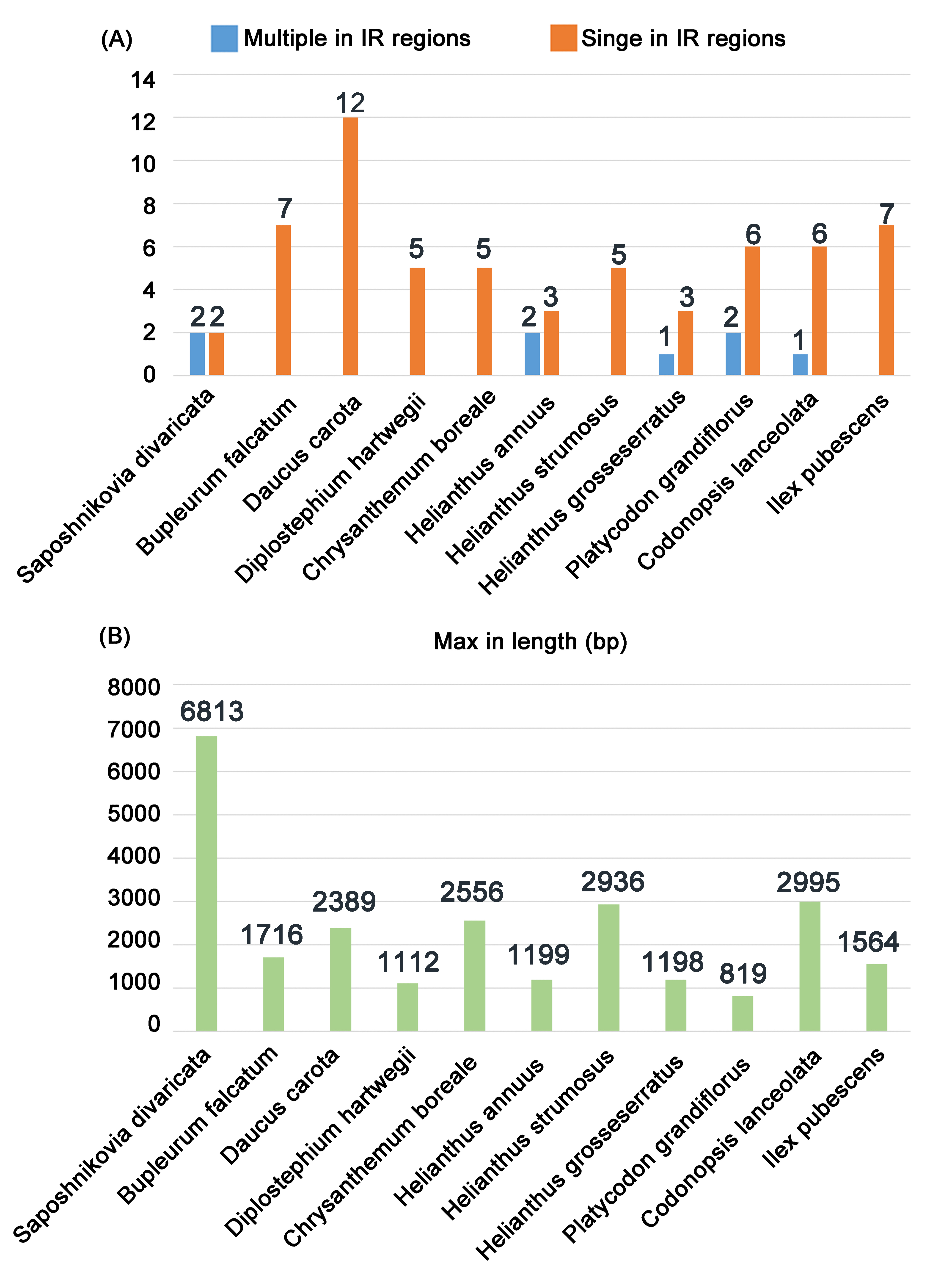


**Supplementary Figure 5. Comparison of the cpgenome and mitogenome sequences suggest the transferring of DNA fragments from the cpgenome to the mitogenome.** (A) The numbers of MTPT in the IR regions. (B) The lengths of the longest MTPTs in Apiales species.
